# Grain Fe and Zn content, heterosis, combining ability and its association with grain yield in irrigated and aerobic rice

**DOI:** 10.1038/s41598-021-90038-4

**Published:** 2021-05-19

**Authors:** G. Anusha, D. Sanjeeva Rao, V. Jaldhani, P. Beulah, C. N. Neeraja, C. Gireesh, M. S. Anantha, K. Suneetha, R. Santhosha, A. S. Hari Prasad, R. M. Sundaram, M. Sheshu Madhav, A. Fiyaz, P. Brajendra, M. D. Tuti, M. H. V. Bhave, K. V. Radha Krishna, J. Ali, D. Subrahmanyam, P. Senguttuvel

**Affiliations:** 1 Indian Institute of Rice Research (ICAR-IIRR), Hyderabad, India; 2 Professor Jaya Shankar Telangana State Agricultural University (PJTSAU), Hyderabad, India; 3 International Rice Research Institute (IRRI), 4031 Los Baños, Laguna Philippines

**Keywords:** Genetics, Plant sciences

## Abstract

Genetic improvement of rice for grain micronutrients, viz*.,* iron (Fe) and zinc (Zn) content is one of the important breeding objectives, in addition to yield improvement under the irrigated and aerobic ecosystems. In view of developing genetic resources for aerobic conditions, line (L) × tester (T) analysis was conducted with four restorers, four CMS lines and 16 hybrids. Both hybrids and parental lines were evaluated in irrigated and aerobic field conditions for grain yield, grain Fe and Zn content. General Combining Ability (GCA) effects of parents and Specific Combining Ability (SCA) effects of hybrids were observed to be contrasting for the micronutrient content in both the growing environments. The grain Fe and Zn content for parental lines were negatively correlated with grain yield in both the contrasting growing conditions. However, hybrids exhibited positive correlation for grain Fe and Zn with grain yield under limited water conditions. The magnitude of SCA mean squares was much higher than GCA mean squares implying preponderance of dominance gene action and also role of complementary non-allelic gene(s) interaction of parents and suitability of hybrids to the aerobic system. The testers HHZ12-SAL8-Y1-SAL1 (T1) and HHZ17-Y16-Y3-Y2 (T2) were identified as good combiners for grain Zn content under irrigated and aerobic conditions respectively.

## Introduction

Rice (*Oryza sativa* L.) is a staple diet and provides more than one-fifth of calories consumed worldwide and also livelihood for more than three billion people in Asia (www.fao.org). It is estimated that over 60% of the world's population are anaemic due to iron (Fe) deficiency and over 30% are zinc (Zn) deficiency^1^, which is referred as hidden hunger. In India 80% of the pregnant women, 52% of the non-pregnant women and 74% of the children (6–35 months) are suffering from Fe deficiency-induced anaemia (IDA)^2^. Rice is mostly transplanted under puddled, fully saturated soil conditions in India. Such soils, almost 50 percent of those soils, are showing signs of Zn deficiency^3^. For India, rice is life and vital primary source of energy. Fortifying such rice’s with high and essential micronutrients will be right step to eliminate “hidden hunger” and significant positive health outcomes for millions of people^4^.

Apart from micronutrient malnutrition, global human population has been steadily increasing over time, leading to demand for further increase in food grain production. The twin issues of food and nutritional security can be addressed through hybrid rice technology. Hybrid rice technology offers an effective way to boost grain yield by exploiting heterosis/hybrid vigour, which is the superiority of F_1_ hybrid over its parents. Adaptation of hybrid rice cultivation has shown 15–20% yield advantage over conventional high-yielding inbred varieties^5^. Even though hybrid technology was commercialised in India during 1994, its area under cultivation has been doubled within a span of 6 years (2016–2020). This shows that there is sudden shift from inbred cultivation to hybrid cultivation and this trend will continue due to availability of good hybrids which are acceptable by farmers.

In view of climate change, depleting water and human resources, exploitation of hybrid rice for aerobic and drought prone ecosystems is the need of the hour for substantial and stabilized yields. Rice crop can adapt to aerobic method of cultivation, since it can tolerate the intermittent water deficit state and high soil impedance. However, various rice genotypes and also hybrids differ in degree of adaptability to aerobic conditions^6^. Hence, identification of suitable genotypes (inbred and parental lines of rice hybrids) and hybrid cross combinations for aerobic ecosystem based on the grain yield and also with higher micronutrient content is the way forward to combat the micronutrient deficiency in the areas where rice is being the major staple diet.

In this regard, understanding the relationship between grain yield and grain micronutrient content under aerobic rice ecosystem will make a best use of heterosis breeding/hybrid rice technology. Although rice grain is known to have low Fe and Zn content, it seems to provide requisite micronutrient supplement to the deprived populace who solely depend on rice and who lack a balanced nutritive diet regularly. Thus, to combat the micronutrient deficiencies among rice eaters, efforts are to be made to enhance the grain micronutrient content (particularly Fe and Zn) along with sustainable crop yield potential. The present investigation was executed to study the combining ability and heterosis in hybrid rice parental lines and their in-house derived hybrid crosses characterized for grain yield, grain Fe and Zn content under aerobic and irrigated methods of cultivation (Tables 1, 2).

**Table 1:** Lines (L), Testers (T) and Hybrids (H) used in L × T mating design study in irrigated and aerobic environments.

**Lines**	
L1	APMS6B
L2	IR68897B
L3	IR79156B
L4	PUSA5B
**Testers**	
T1	HHZ12-SAL8-Y1-SAL1
T2	HHZ17-Y16-Y3-Y2
T3	HHZ2-Y15-Y6-DT1-DT1
T4	HHZ14-Y7-Y1-DT2
**Hybrids/Crosses**	
H1 (L1 × T1)	APMS6A × HHZ12-SAL8-Y1-SAL1
H2 (L1 × T2)	APMS6A × HHZ17-Y16-Y3-Y2
H3 (L1 × T3)	APMS6A × HHZ2-Y15-Y6-DT1-DT1
H4 (L1 × T4)	APMS6A × HHZ14-Y7-Y1-DT2
H5 (L2 × T1)	IR68897A × HHZ12-SAL8-Y1-SAL1
H6 (L2 × T2)	IR68897A × HHZ17-Y16-Y3-Y2
H7 (L2 × T3)	IR68897A × HHZ2-Y15-Y6-DT1-DT1
H8 (L2 × T4)	IR68897A × HHZ14-Y7-Y1-DT2
H9 (L3 × T1)	IR79156A × HHZ12-SAL8-Y1-SAL1
H10 (L3 × T2)	IR79156A × HHZ17-Y16-Y3-Y2
H11 (L3 × T3)	IR79156A × HHZ2-Y15-Y6-DT1-DT1
H12 (L3 × T4)	IR79156A × HHZ14-Y7-Y1-DT2
H13 (L4 × T1)	PUSA-5A × HHZ12-SAL8-Y1-SAL1
H14 (L4 × T2)	PUSA-5A × HHZ17-Y16-Y3-Y2
H15 (L4 × T3)	PUSA-5A × HHZ2-Y15-Y6-DT1-DT1
H16 (L4 × T4)	PUSA-5A × HHZ17-Y16-Y3-Y2
**Checks**	
C1	Sahbhagidhan
C2	CRDhan202

**Table 2:** Soil Characteristics of irrigated and aerobic block of IIRR Farm, Hyderabad.

Block	pH	SOM (%)	Avail N (kg/ha)	Avail P (kg/ha)	Avail K (kg/ha)	Avail Fe (ppm)	Avail Zn (ppm)	Avail Mn (ppm)	Avail Mg (meq/100 g)	Avail Ca (meq/100 g)	Avail S (ppm)	Avail Cu (ppm)	Soil Quality Index Rating
Irrigated (E4 plot)	7.35	1.4	287	19	320	5.30	3.01	5.2	8.0	24	5	8.0	Medium
Direct seeded Aerobic (F9 plot)	7.92	0.93	300	16	280	4.98	2.87	3.0	14.0	28	6	6.65	Medium

## Results

### Performance of hybrids and parental lines

The performance of hybrids and parental lines is presented in Table 3. The results of ANOVA indicates that the genotypic effects for the traits considered are significant (P < 0.01) in both the cultivation methods.

**Table 3:** Mean squares and genetic components for TGW, SPY, Grain Fe and Zn densities in Line × Tester (Including parents) mating design in Irrigated and Aerobic methods. df—Degrees of freedom; *P < 0.05; **P < *0.01.*

Source	df	Irrigated				Aerobic			
		TGW	SPY	Fe	Zn	TGW	SPY	Fe	Zn
Replication	2	0.35	0.78	2.29**	9.72**	0.03	0.04	0.46	9.70*
Genotypes	23	13.15**	14.5**	2.46**	18.08**	9.55**	14.51**	4.12**	53.38**
Cross	15	18.03**	14.17**	1.16**	11.59**	11.95**	12.98**	2.48**	30.85**
Lines (C)	3	26.51**	16.05**	0.71*	6.04**	10.71**	21.24**	3.84**	22.48**
Testers (C)	3	12.5**	1.7	1.21**	26**	6.22**	7.86**	2.18**	91.93**
L × T (C)	9	17.04**	17.7**	1.28**	8.63**	14.27**	11.93**	2.12**	13.29**
Parent	7	4.41**	12.99**	4.28**	28.38**	5.76**	12.53**	7.65**	64.86**
Lines (P)	3	1.47	1.35	6.25**	38.12**	4.02**	2.25	10.88**	63.12**
Testers (P)	3	2.57	3.92**	1.24**	24.5**	0.47	5.07**	1.85**	71.69**
L × T (P)	1	18.7**	75.08**	7.48**	10.8**	26.80**	65.70**	15.36**	49.60**
Cross vs Parent	1	1.16	30.15**	9.25**	43.45**	0.09	51.31**	3.9**	310.93**
Error	46	0.98	0.88	0.19	0.80	0.60	0.81	0.24	3.07
**Proportional contribution of Lines, Testers and Line × Tester mating design**									
Lines		29.42	22.65	12.32	10.43	17.93	32.73	30.97	14.57
Testers		13.87	2.4	20.98	44.88	10.41	12.11	17.63	59.59
Line × Testers		56.71	74.95	66.7	44.69	71.66	55.15	51.4	25.84
**﻿Genetic components**									
σ^2^ GCA		0.034	−0.123	−0.005	0.103	−0.081	0.036	0.012	0.61
σ^2^ SCA		5.418	5.729	0.38	2.589	4.65	3.73	0.59	3.203
σ^2^ GCA / σ^2^ SCA		0.006	−0.021	−0.012	0.04	−0.017	0.01	0.021	0.19
Predictability ratio		0.01	−0.04	−0.02	0.07	−0.04	0.02	0.04	0.28
[2 σ^2^ GCA / (2σ^2^ GCA + σ^2^ SCA)]									

TGW—1000 Grain Weight; SPY—Single Plant Yield; Fe—Grain Fe content; Zn—Grain Zn content.

**Figure 1:**
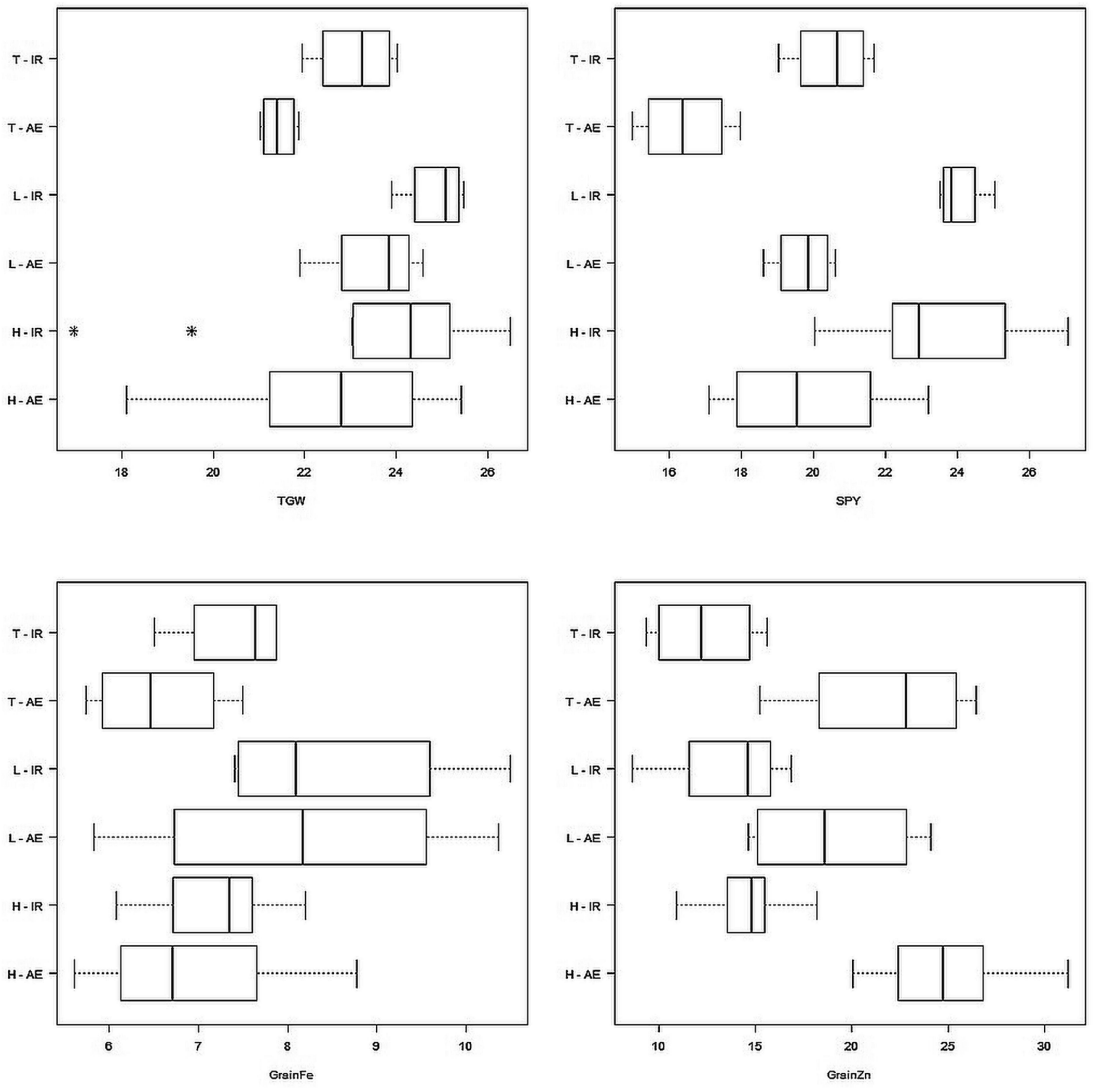
Hybrid and Parental Performance Per se for TGW, SPY, Grain Fe and Grain Zn content. TGW-1000 grain weight (grams); SPY-Single Plant Yield (grams); Grain Fe- Fe content in brown rice (ppm); Grain Zn-Zn content in brown rice (ppm). T-IR-Testers in Irrigated method; T-AE-Testers in Aerobic method; L-IR-Lines in Irrigated method; L-AE-Lines in Aerobic method; H-IR-Hybrids in Irrigated method; H-AE-Hybrids in Aerobic method.

Hybrid and parental per se performance are summarized in Table 4 and Fig. 1. The coefficient of variation (CV) values for the evaluated traits was < 10%, thereby indicating acceptable and effective experiment precision (Table 4). The TGW in hybrids ranged 16.94 g (H3) to 26.50 g (H11) in irrigated method and 18.09 g (H3) to 25.41 g (H11) in aerobic method. The SPY in hybrids ranged 20.04 g (H9) to 27.08 g (H13) in irrigated method and 17.12 g (H4) to 23.21 g (H11) in aerobic method. The mean SPY of hybrids (19.89 g) was higher than that of testers (16.44 g) and lines (19.75 g) in aerobic method. Seven hybrid combinations (H7, H8, H10, H11, H12, H13 and H16) showed higher SPY value than the mean value of hybrids in both the cultivation methods.

**Table 4:** Performance of F_1_﻿ progenies (Hybrids), parent lines (Lines and Testers) and checks for grain yield, TGW, grain Fe and Zn concentration in Irrigated and Aerobic methods.

Entry	Irrigated				Aerobic			
	TGW	SPY	Fe	Zn	TGW	SPY	Fe	Zn
L1	25.25	25.04	7.40	14.70	23.71	20.63	5.83	24.07
L2	23.89	23.53	8.70	8.57	21.89	19.61	7.63	21.60
L3	24.93	23.96	7.50	14.57	24.58	18.61	8.73	14.60
L4	25.48	23.73	10.50	16.87	23.98	20.15	10.37	15.63
T1	24.02	21.70	7.40	13.73	21.66	16.92	7.50	15.23
T2	22.86	20.30	7.87	15.60	21.16	15.90	5.73	21.33
T3	23.67	19.04	6.50	9.30	21.86	14.97	6.83	26.47
T4	21.94	21.07	7.87	10.70	21.03	17.98	6.10	24.37
H1 (L1 × T1)	24.41	22.98	8.03	17.80	23.28	17.53	5.73	20.07
H2 (L1 × T2)	24.27	22.89	7.57	14.97	23.65	18.72	5.80	26.53
H3 (L1 × T3)	16.94	21.95	6.87	14.07	18.09	19.35	6.23	22.50
H4 (L1 × T4)	23.06	21.20	7.43	14.60	21.44	17.12	6.60	23.13
H5 (L2 × T1)	24.74	22.43	6.47	15.50	19.79	18.03	7.73	22.37
H6 (L2 × T2)	23.38	22.87	7.77	15.43	21.68	19.45	7.67	26.37
H7 (L2 × T3)	24.90	23.92	8.20	16.73	24.18	21.03	5.60	26.50
H8 (L2 × T4)	25.93	24.54	6.77	13.00	24.57	20.37	6.87	23.97
H9 (L3 × T1)	23.07	20.04	7.57	15.27	20.87	17.35	6.03	20.03
H10 (L3 × T2)	25.43	27.04	7.63	14.03	24.54	22.84	8.00	31.23
H11 (L3 × T3)	26.50	26.99	6.07	12.40	25.41	23.21	6.40	27.10
H12 (L3 × T4)	25.98	26.12	6.33	14.33	24.59	22.12	7.07	21.77
H13 (L4 × T1)	19.52	27.08	7.50	18.17	22.28	22.65	8.77	23.13
H14 (L4 × T2)	23.84	22.10	7.27	12.13	23.00	17.73	6.83	28.27
H15 (L4 × T3)	23.03	22.32	6.67	15.13	22.59	19.65	6.60	25.50
H16 (L4 × T4)	24.77	24.21	7.17	10.87	20.99	21.04	7.63	28.67
Check-C1	25.79	25.03	7.37	20.33	25.38	24.45	7.10	29.13
Check-C2	25.77	26.04	9.87	20.41	25.49	24.85	9.27	23.60
Mean (Lines)	24.89	24.07	8.53	13.68	23.54	19.75	8.14	18.98
Mean (Testers)	23.12	20.53	7.41	12.33	21.43	16.44	6.54	21.85
Mean (Parents)	24.01	22.30	7.97	13.01	22.48	18.10	7.34	20.41
Mean (Crosses)	23.74	23.67	7.21	14.65	22.56	19.89	6.85	24.82
Mean (Total)	23.83	23.21	7.46	14.10	22.53	19.29	7.01	23.35
CV (%)	4.17	4.05	5.85	6.34	3.43	4.67	7.02	7.51
SE	0.57	0.54	0.25	0.52	0.45	0.52	0.28	1.01
SED	0.81	0.77	0.36	0.73	0.63	0.74	0.40	1.43
CD(P = 05)	1.63	1.54	0.72	1.47	1.27	1.48	0.81	2.88
CD(P = 01)	2.17	2.06	0.95	1.96	1.69	1.97	1.08	3.84

TGW—1000 Grain Weight; SPY—Single Plant Yield; Fe—Grain Fe content; Zn—Grain Zn content; L1 to L4—Lines; T1 to T4—Testers; H1 to H16—Hybrids.

The grain Zn concentration of hybrids ranged from 10.87 ppm to 17.80 ppm and 20.03 ppm to 31.23 ppm in irrigated and aerobic methods respectively. All the hybrids recorded higher grain Zn concentration; the mean of grain Zn concentration of lines (38%), testers (77%) and hybrids (69%) was higher under aerobic method of cultivation.

### Heterosis

Three categories of heterosis, viz*.,* standard heterosis (SH), Mid parent heterosis (MPH) and Better parent heterosis (BPH) exhibited significant variation in both the cultivation methods (Tables 5, 6, 7 and Supplementary Figs. 1, 2, 3, 4). Of these, MPH recorded the higher estimated heterosis values for TGW, SPY and grain Zn concentration. While grain Fe concentration recorded highest average SH values in both the cultivation methods. More than 50% of the estimated values for the crosses showed positive MPH in both the cultivation methods.

**Table 5:** Estimation of mid-parent heterosis (MPH) for yield components (TGW and SPY), grain Fe and Zn concentration of crosses (Hybrids) in irrigated and aerobic method. *P < 0.05; **P < *0.01; ns—not significant.*

Entry	Irrigated				Aerobic			
	TGW	SPY	Fe	Zn	TGW	SPY	Fe	Zn
H1 (L1 × T1)	−0.89 ns	−1.64 ns	8.56 *	25.21 **	2.62 ns	−6.61 ns	−14 **	2.12 ns
H2 (L1 × T2)	0.91 ns	0.99 ns	−0.87 ns	−1.21 ns	5.39 *	2.46 ns	0.29 ns	16.89 **
H3 (L1 × T3)	−30.75 **	−0.42 ns	−1.2 ns	17.22 **	−20.62 **	8.73 *	−1.58 ns	−10.95 *
H4 (L1 × T4)	−2.28 ns	−8.03 **	−2.62 ns	14.96 **	−4.16 ns	−11.32 **	10.61 ns	−4.47 ns
Mean	−8.25	−2.28	0.97	14.05	−4.19	−1.69	−1.17	0.90
H5 (L2 × T1)	3.26 ns	−0.79 ns	−19.67 **	39.01 **	−9.11 **	−1.3 ns	2.2 ns	21.45 **
H6 (L2 × T2)	0 ns	4.35 ns	−6.24 ns	27.72 **	0.73 ns	9.55 *	14.71 **	22.83 **
H7 (L2 × T3)	4.7 ns	12.4 **	7.89 ns	87.31 **	10.52 **	21.62 **	−22.58 **	10.26 ns
H8 (L2 × T4)	13.16 **	10.05 **	−18.31 **	34.95 **	14.47 **	8.37 *	0 ns	4.28 ns
Mean	5.28	6.5025	−9.0825	47.2475	4.1525	9.56	−1.4175	14.705
H9 (L3 × T1)	−5.75 ns	−12.21 **	1.57 ns	7.89 ns	−9.76 **	−2.35 ns	−25.67 **	34.3 **
H10 (L3 × T2)	6.44 *	22.2 **	−0.65 ns	−6.96 ns	7.28 **	32.39 **	10.6 *	73.84 **
H11 (L3 × T3)	9.06 **	25.53 **	−13.33 **	3.91 ns	9.4 **	38.27 **	−17.77 **	31.98 **
H12 (L3 × T4)	10.87 **	16.01 **	−17.57 **	13.46 **	7.83 **	20.88 **	−4.72 ns	11.72 ns
Mean	5.16	12.88	−7.50	4.58	3.69	22.30	−9.39	37.96
H13 (L4 × T1)	−21.14 **	19.22 **	−16.2 **	18.74 **	−2.39 ns	22.21 **	−1.87 ns	49.89 **
H14 (L4 × T2)	−1.38 ns	0.39 ns	−20.87 **	−25.26 **	1.91 ns	−1.62 ns	−15.11 **	52.93 **
H15 (L4 × T3)	−6.28 *	4.36 ns	−21.57 **	15.67 **	−1.45 ns	11.91 **	−23.26 **	21.14 **
H16 (L4 × T4)	4.48 ns	8.07 **	−21.96 **	−21.16 **	−6.72 **	10.35 **	−7.29 ns	43.33 **
Mean	−6.08	8.01	−20.15	−3.00	−2.16	10.71	−11.88	41.82
Total Mean	−0.97	6.28	−8.94	15.72	0.37	10.22	−5.97	23.85

TGW—1000 Grain Weight; SPY—Single Plant Yield; Fe—Grain Fe content; Zn﻿—Grain Zn content; L1 to L4—Lines; T1 to T4—Testers; H1 to H16 – Hybrids.

**Table 6:** Estimation of best-parent heterosis (BPH) for yield components (TGW and SPY), grain Fe and Zn concentration of crosses (Hybrids) in irrigated and aerobic method. *P < 0.05; **P < *0.01; ns—not significant.*

Entry	Irrigated				Aerobic			
	TGW	SPY	Fe	Zn	TGW	SPY	Fe	Zn
H1 (L1 × T1)	−3.31 ns	−8.2 *	8.56 ns	21.09 **	−1.81 ns	−15.02 **	−23.56 **	−16.62 **
H2 (L1 × T2)	−3.87 ns	−8.56 **	−3.81 ns	−4.06 ns	−0.28 ns	−9.29 *	−0.57 ns	10.25 ns
H3 (L1 × T3)	−32.91 **	−12.34 **	−7.21 ns	−4.31 ns	−23.71 **	−6.2 ns	−8.78 ns	−14.99 **
H4 (L1 × T4)	−8.69 **	−15.32 **	−5.51 ns	−0.68 ns	−9.59 **	−17.01 **	8.2 ns	−5.06 ns
Mean	−12.20	−11.11	−1.99	3.01	−8.85	−11.88	−6.18	−6.61
H5 (L2 × T1)	3 ns	−4.65 ns	−25.67 **	12.86 *	−9.59 **	−8.07 *	1.31 ns	3.55 ns
H6 (L2 × T2)	−2.16 ns	−2.81 ns	−10.73 *	−1.07 ns	−0.96 ns	−0.82 ns	0.44 ns	22.07 **
H7 (L2 × T3)	4.21 ns	1.69 ns	−5.75 ns	79.93 **	10.44 **	7.22 ns	−26.64 **	0.13 ns
H8 (L2 × T4)	8.54 *	4.29 ns	−22.22 **	21.5 **	12.21 **	3.88 ns	−10.04 ns	−1.64 ns
Mean	3.40	−0.37	−16.09	28.31	3.03	0.55	−8.73	6.03
H9 (L3 × T1)	−7.47 *	−16.35 **	0.89 ns	4.81 ns	−15.12 **	−6.79 ns	−30.92 **	31.51 **
H10 (L3 × T2)	2.02 ns	12.87 **	−2.97 ns	−10.04 *	−0.19 ns	22.75 **	−8.4 ns	46.41 **
H11 (L3 × T3)	6.31 ns	12.65 **	−19.11 **	−14.87 **	3.35 ns	24.74 **	−26.72 **	2.39 ns
H12 (L3 × T4)	4.23 ns	9.02 **	−19.49 **	−1.6 ns	0.04 ns	18.84 **	−19.08 **	−10.67 ns
Mean	1.27	4.55	−10.17	−5.43	−2.98	14.89	−21.28	17.41
H13 (L4 × T1)	−23.4 **	14.1 **	−28.57 **	7.71 ns	−7.1 **	12.41 **	−15.43 **	47.97 **
H14 (L4 × T2)	−6.45 *	−6.87 *	−30.79 **	−28.06 **	−4.09 ns	−11.99 **	−34.08 **	32.5 **
H15 (L4 × T3)	−9.6 **	−5.96 ns	−36.51 **	−10.28 *	−5.8 *	−2.48 ns	−36.33 **	−3.65 ns
H16 (L4 × T4)	−2.77 ns	1.99 ns	−31.75 **	−35.57 **	−12.45 **	4.42 ns	−26.37 **	17.65 **
Mean	−10.56	0.82	−31.91	−16.55	−7.36	0.59	−28.05	23.62
Total Mean	−4.52	−1.53	−15.04	2.34	−4.04	1.04	−16.06	10.11

TGW—1000 Grain Weight; SPY—Single Plant Yield; Fe—Grain Fe content; Zn—Grain Zn content; L1 to L4—Lines; T1 to T4—Testers; H1 to H16—Hybrids.

**Table 7:** Estimation of standard heterosis (SH) for yield components (TGW and SPY), grain Fe and Zn concentration of crosses (Hybrids) in irrigated and aerobic method. *P < 0.05; **P < *0.01; ns—not significant.*

Entry	Irrigated				Aerobic			
	TGW	SPY	Fe	Zn	TGW	SPY	Fe	Zn
H1 (L1 × T1)	−5.35 ns	−8.18 **	9.05 ns	−12.46 **	−8.26 **	−28.29 **	−19.25 **	0.43 ns
H2 (L1 × T2)	−5.89 *	−8.54 **	2.71 ns	−26.39 **	−6.83 **	−23.45 **	−18.31 **	0.2 ns
H3 (L1 × T3)	−34.32 **	−12.32 **	−6.79 ns	−30.82 **	−28.72 **	−20.85 **	−12.21 ns	−1.14 ns
H4 (L1 × T4)	−10.61 **	−15.3 **	0.9 ns	−28.2 **	−15.52 **	−29.97 **	−7.04 ns	0.51 ns
Mean	−14.04	−11.09	1.47	−24.47	−14.83	−25.64	−14.20	0.00
H5 (L2 × T1)	−4.1 ns	−10.37 **	−12.22 *	−23.77 **	−22.01 **	−26.27 **	8.92 ns	0.99 ns
H6 (L2 × T2)	−9.37 **	−8.64 **	5.43 ns	−24.1 **	−14.57 **	−20.45 **	7.98 ns	−1.71 ns
H7 (L2 × T3)	−3.46 ns	−4.42 ns	11.31 ns	−17.7 **	−4.73 *	−14 **	−21.13 **	1.12 ns
H8 (L2 × T4)	0.54 ns	−1.97 ns	−8.14 ns	−36.07 **	−3.2 ns	−16.69 **	−3.29 ns	−0.4 ns
Mean	−4.10	−6.35	−0.91	−25.41	−11.13	−19.35	−1.88	0.00
H9 (L3 × T1)	−10.57 **	−19.94 **	2.71 ns	−24.92 **	−17.78 **	−29.05 **	−15.02 *	−1.58 ns
H10 (L3 × T2)	−1.4 ns	8.03 **	3.62 ns	−30.98 **	−3.32 ns	−6.57 *	12.68 ns	2.92 *
H11 (L3 × T3)	2.75 ns	7.82 **	−17.65 **	−39.02 **	0.11 ns	−5.06 ns	−9.86 ns	1.49 ns
H12 (L3 × T4)	0.74 ns	4.34 ns	−14.03 *	−29.51 **	−3.1 ns	−9.54 **	−0.47 ns	−2.83 *
Mean	−2.12	0.06	−6.34	−31.11	−6.02	−12.56	−3.17	0.00
H13 (L4 × T1)	−24.33 **	8.19 **	1.81 ns	−10.66 **	−12.23 **	−7.36 **	23.47 **	0.16 ns
H14 (L4 × T2)	−7.59 **	−11.69 **	−1.36 ns	−40.33 **	−9.38 **	−27.47 **	−3.76 ns	−1.4 ns
H15 (L4 × T3)	−10.7 **	−10.83 **	−9.5 ns	−25.57 **	−10.99 **	−19.63 **	−7.04 ns	−1.47 ns
H16 (L4 × T4)	−3.95 ns	−3.29 ns	−2.71 ns	−46.56 **	−17.28 **	−13.95 **	7.51 ns	2.71 *
Mean	−11.64	−4.41	−2.94	−30.78	−12.47	−17.10	5.05	0.00
Total Mean	−7.98	−5.44	−2.18	−27.94	−11.11	−18.66	−3.55	0.00

TGW—1000 Grain Weight; SPY—Single Plant Yield; Fe—Grain Fe content; Zn—Grain Zn content; L1 to L4—Lines; T1 to T4—Testers; H1 to H16—Hybrids.

For TGW, four F_1_ progenies—H8, H10, H11 and H12 recorded significant positive estimates for MPH in both cultivation methods. Among these, H8 and H11 were observed with higher MPH in aerobic method. In addition, H8 had highest and consistent significant positive estimates for both MPH (Irrigated—13.16%; Aerobic—14.47%) and BPH (Irrigated—8.54%; Aerobic—12.21%) among all the crosses in both the cultivation methods. In other words, MPH was marginally higher while BPH was nearly 43% higher in aerobic method than irrigated method. However, the higher percentage of crosses showed positive MPH in aerobic method than irrigated for all the traits studied except for SPY for which it was remains unchanged. Although six F_1_ progenies—H7, H10, H11, H12, H13 and H16 showed significant *(P* < *0.01)* positive MPH for SPY in both the cultivation methods, higher MPH values were observed for aerobic method. In addition, four F_1_ progenies—H10, H11, H12 and H13 showed consistent significant positive estimates for both MPH and BPH for SPY.

For Fe concentration, H7 and H13 had the higher positive estimates (but not significant) for SH in irrigated and aerobic methods respectively. Over all, the F_1_ progenies, H6, H10 and H13 were observed with positive estimates for SH in both the cultivation methods. Interestingly, H10, H11 and H12 recorded significant positive MPH estimates for yield traits (SPY and TGW) in both the cultivation methods. H13 had showed a positive MPH estimates for SPY and grain Zn concentration; in contrast it had a negative MPH estimate for grain Fe concentration.

For Zn concentration, 10 F_1_ progenies had consistent significant positive estimates for the MPH in irrigated (H7 > H5 > H8 > H6 > H1 > H13 > H3 > H15 > H4 > H12) and aerobic (H10 > H14 > H13 > H16 > H9 > H11 > H6 > H5 > H15 > H2) cultivation methods. Among them, four crosses—H5, H6, H13 and H15 were common in both the methods and two crosses (H13 and H15) had higher significant positive estimates in aerobic method. In addition, the crosses H7 (87.31%) and H10 (73.84%) were observed with higher significant positive estimates of MPH for grain Zn in irrigated and aerobic methods respectively. Particularly in irrigated method, four F_1_ progenies (H1, H5, H7 and H8) had significant positive estimates for both BPH and MPH; whereas, the six crosses (H6, H9, H10, H13, H14 and H16) had significant positive estimates for both BPH and MPH in aerobic method.

### Combining ability

The ANOVA for the L × T mating design is summarized in Table 3. Variance due to parents, genotype, crosses, lines (c), testers (c) and line × testers (c) were significant (P < 0.01) for all traits in both the cultivation methods. Variances due to crosses versus parents were significant for all traits with the exception for TGW in both the conditions. Proportionate contribution to the total variance by lines, testers and L × T revealed that L × T had contributed the most with respect to all of the characterized traits except for grain Zn concentration for which testers have contributed the most in both the cultivation methods.

### GCA effect of parents

No R-line and B-line that concurrently had positive GCA effects for all the studied traits in both cultivation methods (Table 8 and Supplementary Figs. 5, 6, 7, 8). Mostly, 50% of the testers had positive GCA effect for all traits studied in both the cultivation methods except for grain Zn concentration in irrigated method. For SPY, three R-lines (T4 > T3 > T2) had positive GCA effect (non-significant) in irrigated method; T1 had significant negative GCA effect for SPY and TGW; positive GCA effect for grain Fe concentration in both the cultivation methods. However, grain Zn concentration for the same tester (T1) had significant GCA effect but positive in irrigated (2.03**) and negative in aerobic (−3.42**) method. T1 is merely tester with significant positive GCA effect (2.03**) for grain Zn concentration in irrigated method. But in aerobic method, T2 had the significant positive GCA effect (3.28**) for grain Zn concentration. The tester (T4) with positive GCA effect for yield traits had negative GCA effect for grain Zn concentration in both the cultivation methods. Likewise, the tester (T1) with negative GCA effect for yield traits had positive GCA effect for grain Fe concentration in both the cultivation methods. The GCA effect of grain Zn concentration in testers—T1, T2 and T3 was observed with converse pattern in the cultivation methods *i.e.,* the tester with positive GCA effect in irrigated method, had negative GCA effect in aerobic method and vice-versa. However, the GCA effects of grain Fe concentration for the same testers maintain the same pattern (positive or negative) in both the cultivation methods.

**Table 8:** Estimation of relative general combining ability (GCA) effects of parents, relative specific combining ability (SCA) effects of crosses (Hybrids) for TGW, SPY, grain Fe and Zn concentration in Irrigated and Aerobic methods.*P < 0.05; **P < *0.01; ns—not significant.*

	Entry	Irrigated				Aerobic			
		TGW	SPY	Fe	Zn	TGW	SPY	Fe	Zn
LINES	L1	−1.57 **	−1.41 **	0.27 *	0.71 *	−0.94 **	−1.71 **	−0.76 **	−1.76 **
	L2	1 **	−0.23 ns	0.09 ns	0.51 ns	0 ns	−0.17 ns	0.12 ns	−0.02 ns
	L3	1.51 **	1.38 **	−0.31 **	−0.64 *	1.29 **	1.49 **	0.03 ns	0.21 ns
	L4	−0.95 **	0.26 ns	−0.06 ns	−0.58 *	−0.34 *	0.38 ns	0.61 **	1.57 **
	SE of LINES	0.26	0.29	0.11	0.27	0.16	0.25	0.17	0.55
TESTERS	T1	−0.8 **	−0.53 *	0.19 ns	2.03 **	−1 **	−1 **	0.22 ns	−3.42 **
	T2	0.49 ns	0.06 ns	0.35 **	−0.51 ns	0.66 **	−0.2 ns	0.23 ns	3.28 **
	T3	−0.89 **	0.13 ns	−0.26 *	−0.07 ns	0.01 ns	0.92 **	−0.64 **	0.58 ns
	T4	1.2 **	0.35 ns	−0.28 *	−1.45 **	0.34 *	0.28 ns	0.19 ns	−0.44 ns
	SE of TESTERS	0.26	0.29	0.11	0.27	0.16	0.25	0.17	0.55
HYBRIDS/CROSSES	H1 (L1 × T1)	3.05 **	1.26 **	0.37 ns	0.41 ns	2.67 **	0.35 ns	−0.58 ns	0.43 ns
	H2 (L1 × T2)	1.61 **	0.58 ns	−0.26 ns	0.12 ns	1.37 **	0.74 ns	−0.52 ns	0.2 ns
	H3 (L1 × T3)	−4.34 **	−0.44 ns	−0.35 ns	−1.22 *	−3.53 **	0.25 ns	0.78 *	−1.14 ns
	H4 (L1 × T4)	−0.31 ns	−1.4 **	0.24 ns	0.69 ns	−0.51 ns	−1.33 *	0.31 ns	0.51 ns
	H5 (L2 × T1)	0.8 ns	−0.47 ns	−1.02 **	−1.7 **	−1.76 **	−0.69 ns	0.55 ns	0.99 ns
	H6 (L2 × T2)	−1.85 **	−0.63 ns	0.11 ns	0.78 ns	−1.53 **	−0.07 ns	0.47 ns	−1.71 ns
	H7 (L2 × T3)	1.06 *	0.36 ns	1.16 **	1.64 **	1.62 **	0.38 ns	−0.73 *	1.12 ns
	H8 (L2 × T4)	0 ns	0.75 ns	−0.25 ns	−0.71 ns	1.67 **	0.38 ns	−0.29 ns	−0.4 ns
	H9 (L3 × T1)	−1.38 *	−4.47 **	0.48 *	−0.77 ns	−1.98 **	−3.04 **	−1.06 **	−1.58 ns
	H10 (L3 × T2)	−0.31 ns	1.94 **	0.38 ns	0.54 ns	0.03 ns	1.66 **	0.9 *	2.92 *
	H11 (L3 × T3)	2.15 **	1.81 **	−0.58 *	−1.54 **	1.55 **	0.91 ns	0.16 ns	1.49 ns
	H12 (L3 × T4)	−0.46 ns	0.72 ns	−0.29 ns	1.78 **	0.4 ns	0.46 ns	0 ns	−2.83 *
	H13 (L4 × T1)	−2.47 **	3.69 **	0.16 ns	2.06 **	1.07 **	3.38 **	1.09 **	0.16 ns
	H14 (L4 × T2)	0.55 ns	−1.88 **	−0.24 ns	−1.43 *	0.13 ns	−2.33 **	−0.85 *	−1.4 ns
	H15 (L4 × T3)	1.14 *	−1.73 **	−0.23 ns	1.13 *	0.37 ns	−1.54 **	−0.22 ns	−1.47 ns
	H16 (L4 × T4)	0.78 ns	−0.07 ns	0.3 ns	−1.76 **	−1.56 **	0.5 ns	−0.02 ns	2.71 *
	SE of crosses	0.51	0.59	0.22	0.54	0.33	0.50	0.34	1.11

TGW—1000 Grain Weight; SPY—Single Plant Yield; Fe—Grain Fe content; Zn—Grain Zn content; L1 to L4—Lines; T1 to T4—Testers; H1 to H16—Hybrids.

### SCA effect of crosses

Similar to the results noticed for GCA effects of parents, there was no hybrid cross that concurrently attained positive SCA effects for all of the studied traits (Table 8 and Supplementary Figs. 9, 10, 11, and 12). Nearly, 50% of the hybrid combinations had positive SCA effect for all traits studied in both the cultivation methods. However, the percentage of hybrid crosses with positive SCA effect was higher in aerobic than irrigated method for yield traits. For grain Fe and Zn concentration the percentage of crosses with positive SCA effect remains constant in both the methods, though the individual crosses showed differential response. The hybrids (H13 > H10 > H11 > H1) had showed significant (P < 0.01) positive SCA effect for SPY in irrigated method. For SPY, the hybrid combinations H10 and H13 had showed significant positive SCA effect in both the cultivation methods. Moreover, H13 had highest positive SCA effect for SPY (3.69**) and grain Zn concentration (2.06**) in irrigation method; conversely in aerobic method, it had highest positive SCA effect for SPY (3.38**) and grain Fe concentration (1.09**) in aerobic method. A few hybrid combinations with negative SCA effect in irrigation method showed positive SCA effect in aerobic method. For instance, the hybrid combinations—H3 and H16 for SPY; H10, H12 and H13 for TGW; H3, H5 and H11 for grain Fe concentration; H5, H11 and H16 for grain Zn concentration showed negative SCA effect in irrigated method and positive SCA effect in aerobic method. Some hybrid combinations showed improvement in their positive SCA effects from irrigated method to aerobic method. For instance, the hybrid combinations—H7 for SPY; H7 and H8 for TGW; H4, H6, H10 and H13 for grain Fe concentration; H1, H2 and H10 for grain Zn concentration showed an improvement in their positive SCA effect from irrigated to aerobic method. In addition, the variance due to SCA (σ^2^ SCA) was greater than GCA (σ^2^ GCA) for all traits studied (Table 3). In the present study, the GCA values of eight parental were used to calculate GSCA values of 16 crosses. The relative GSCA effects of crosses for yield components and grain Fe and Zn concentration in irrigated and aerobic methods were also estimated (Supplementary Table 1).

### Correlation between phenotype, heterosis and combining ability

The association between phenotype, heterosis and combining ability effects for yield components, grain Fe and Zn concentrations in irrigated and aerobic methods was represented in Supplementary Table 2. GCA did not have any significant correlation with SCA effects, SH, BPH and phenotype for all traits in both the cultivation methods. For MPH, GCA had significant relationship with SPY (r = 0.62) in irrigated condition. SCA had positive significant (*P* < *0.001*) correlations with SH, MPH and BPH for yield component traits and grain Fe and Zn concentration in both the cultivation methods. Negative correlation between SCA and phenotype was observed for yield traits and grain FE concentration in both cultivation methods. However, the grain Zn concentration (r = 0.54) had significant positive relationship with SCA effect in aerobic method. GSCA had significant (*P* < *0.01*) positive association with SCA, SH, MPH and BPH of SPY. For grain Fe and Zn concentration in irrigation method, the GSCA had significant (*P* < *0.01*) positive association with GCA effect. On the contrary, it had negative interaction with SCA, SH, MPH and BPH. In aerobic method, the GSCA had positive interaction with SCA, SH, MPH and BPH of SPY and grain Fe and Zn concentration. In addition, the association between GCA effect and GSCA of SPY and grain Zn concentration was positively significant.

The association among the traits of parents and hybrids in irrigated and aerobic methods is represented in Supplementary Table 3. The correlation between grain Fe and Zn concentration was significantly positive (r = 0.50, *P* < *0.01*) in hybrids under irrigated method; in contrast significantly negative (r = -0.73, *P* < *0.01*) in parents under aerobic method. However, both micronutrients had significant and moderate positive association with SPY (r = 0.43 (Fe) and 0.42 (Zn), *P* < *0.01*) in hybrids under aerobic method but weak negative correlation (r =  − 0.23 (Fe) and − 0.05 (Zn)) in irrigated method.

## Discussion

Rice is cultivated over a wide range of agro-climatic conditions and is one of the most consumed crops worldwide, especially in developing countries (http://ricepedia.org/rice-as-a-crop). However, sustainable rice production suffers major setback due to non-availability of fresh water and insufficient genetic gain. Systematic breeding to increase grain yield, which is also fortified with high micronutrient content in grain under limited water condition is top priority areas of rice research under climate change scenario.

In recent years, rice cultivation is in transition from traditional methods of water-logged fields to water saving cultivation methods like SRI and “aerobic rice” due to water resource constraints in the context of a rapidly changing climate^7,8^. So far, rice breeding has primarily focused on realizing targeted crop yield returns in both irrigated and aerobic method. However, with the emphasis on sustaining global food and nutritional security, studies on essential micronutrients (grain Fe and Zn content) enhancement has to be prioritized. The essential micronutrient content is poor in rice grain and cannot meet the daily human nutritional requirements in contrast to other cereals^9^. Zinc deficiency, especially in the rice based diet among consumers of developing countries, is being addressed through development of biofortified rice with high zinc in polished rice^10^. Significant progress has been achieved in developing high Zn rice varieties suitable for cultivation in irrigated ecosystems. Biofortified rice varieties like DRR Dhan 45, Chhattisgarh Zinc Rice-1 (CGZR-1), Chhattisgarh Zinc Rice-2 (CGZR-2), Zinco Rice-MS, BRRI Dhan 62, BRRI Dhan 64, BRRI Dhan 72, DRR Dhan 48, DRR Dhan 49, CR Dhan 315 and Surabhi with high Zn content in polished rice were released for commercial cultivation in different countries. Several studies are available on Zn Bio-fortification in rice under irrigated methods^10–12^. However, little progress in yield improvement and Bio-fortification has been made under aerobic condition.

In the present study, using L × T design, grain yield, grain Fe and Zn content were analysed in hybrids and their parental lines grown under aerobic and irrigated conditions. In our study, some hybrids performed better for SPY and TGW traits than their parents and check variety and exhibited the marked hybrid vigour in both the cultivation methods. Our observations are concurrent with previous results as SPY had significant correlations with TGW^13,14^^.^ Three categories of estimated heterosis values shown varying degrees for MPH, BPH and SH for the studied traits. The observed results are in harmony with earlier reports^14–18^. Among the three categories of heterosis, MPH (3.02 in irrigated; 7.12 in aerobic) had the highest average values for all the studied traits, followed by BPH (-4.69 in irrigated; -2.24 in aerobic) and SH (-10.89 in irrigated; -8.33 in aerobic) in both the cultivation methods. These observations are similar to the previous studies^14,15^. Basically, SH is the most appropriate among the three categories of heterosis. SH elucidated the acceptable yield advantage (20–30%) of hybrid varieties over the best inbred varieties. The hybrids in the present study are observed with average negative heterosis for all the studied traits in both irrigated and aerobic methods.

The GCA elucidates the average performance of a line in an array of hybrid crosses and it can be used to differentiate the parental lines In our study, the GCA effect of testers (except T4) for grain Zn content is in contrasting manner with the cultivation methods. In irrigated method, the GCA effect of SPY and grain Fe and Zn contents were observed in contrasting manner. Negative correlation and negative GCA effect was observed between grain yield and Fe & Zn content under irrigated and aerobic method indicates the need for Fe and Zn content improvement of parental lines in line with grain yield^19^.

SCA is mainly associated with non-additive gene action resulting from dominance, over dominance and epistatic effects^20–24^. Analysis of the hybrids revealed that no specific combination obtained significant positive SCA values for all of the estimated traits simultaneously. This result is in agreement with those from previous studies in rice^14,16,18,23,25^ as well as in maize^26^, bread wheat^27^. However, the crosses H1 [significant (*P* < *0.01*) for yield traits] and H7 [significant (*P* < *0.01*) for grain Fe and Zn content] showed positive SCA values for all the traits in irrigated method. Conversely, in aerobic method—H10 (significant for SPY and grain Fe and Zn content), H11 (TGW) and H13 (TGW, SPY and grain Fe content) showed positive SCA values for all the traits. These results were in agreement with previous studies in rice^14,28^, pearl millet^29,30^ and maize^31,32^ indicating that the grain micronutrient concentrations are mostly under additive genetic control.

The parents of cross combination with significant SCA effect for a particular trait, may not possess significant GCA effects. For instance, the cross H1 had significant positive SCA effect for yield traits in irrigated method and its parents had significant negative GCA effects. There were no significant SCA effects in all crosses for the estimated traits, suggesting that the values for these traits are within the limits of the parent’s averages. Interestingly, parents with significant GCA effects did not result the best hybrids with significant SCA. For instance, the parents of H2 and H16 showed positive GCA effects, but hybrids were observed with negative SCA effects for grain Fe content in irrigated and aerobic methods respectively. This observation could be attributed to different combination of dominant and recessive genes or alleles in repulsion phase from one of the parents; it further substantiates the operation of non-additive gene action (additive × dominance, dominance × dominance and epistatic interactions). The hybrid H13 was observed with highest significant positive SCA effect for SPY and grain Zn content in irrigated method; SPY and grain Fe content in aerobic method. For TGW trait, the hybrid H1 had highest significant positive SCA effect in both the cultivation methods, in spite of both parents were with negative GCA effect. These observations are similar to the previous studies^16–18,23,25^. Generally, the hybrids with high SCA effects are recommended for heterosis breeding.

The association of GCA with per se performance and heterotic effects was poor since most of the traits were under non-additive gene action. The inheritance of quantitative traits like yield involves complex mechanisms. Thus, the GCA effects of parents are inadequate to predict the SCA effects of respective hybrids^24,33,34^. Conversely, the significant correlation between SCA effects and heterosis was observed for all studied traits in both the cultivation methods. In general, the GCA effects of parents can be taken as basis for coupling the hybrid combinations in heterosis breeding targeted for a particular trait^18,35^. However, the parents with low GCA effects were also considered like as in maize^36^. The sum of GCAs of parental lines of hybrids represents the GSCA (General Sum of Combining Ability) value^18^. GSCA had a positive association with SCA and heterosis traits for SPY in irrigated condition; SPY, grain Fe and Zn content in aerobic condition. These results are similar to Huang et al^18^ and contrast with Gramaje et al^14^. The positive correlation between GSCA and phenotype was observed for grain Zn content in aerobic method as in case of Gramaje et al^14^. The concentration of the most of the nutrient elements in soil increases on submergence, but the availability of Zn to plant decreases^37^. The increased Zn content in grains of most of the entries grown under aerobic conditions indicates that there is a greater mobilization of zinc from source to sink under aerobic conditions^38^. Apart from genetic variability, agronomic management practices such as spraying of ZnSo4@4 g. Litre^-1^ at early vegetative stage under aerobic conditions also plays a significant role in accumulation of more Zn in rice grain.

Positive correlation between grain Fe and Zn concentration was observed in parents as well as in the hybrids in irrigated method. Similar associations between these grain micronutrient concentrations have been reported previously in rice^14,39,40^, maize^41–43^, wheat^44–46^, sorghum^47,48^, pearl millet^30,49–51^ and finger millet^52^. To the best of our knowledge, this is the first report on evaluation of rice parental lines, hybrids under aerobic and flooded conditions for grain micronutrient content.

## Conclusions

The study elucidates variation in Fe and Zn content in relation with grain yield under aerobic and irrigated conditions. Though many studies are not available in estimation of micronutrient content under aerobic conditions, the present findings clearly indicate that the hybrids exhibited higher micronutrient content coupled with grain yield under aerobic than transplanted irrigated conditions. The study paved way for further improvement of Fe and Zn content under aerobic system and also large scale adoption of aerobic rice cultivation in water limited conditions**.**

## Materials and methods

### Plant materials and field performance evaluation

The aerobic adapted promising parental lines were selected based on the previous studies^53,54^ and were validated for their yield performance and micronutrient content under aerobic and irrigated conditions. A total of 16 test crosses were developed by following Line × Tester mating design^55^ using four restorer and four CMS lines. The hybrids (Crosses), testers (T—R lines/Male parents), lines (L—CMS lines/Female parents) and varietal checks, CR Dhan 202 (C1) and Shabhagidhan (C2) were evaluated at Research Farm of ICAR-Indian Institute of Rice Research (ICAR-IIRR), Hyderabad, India (17º 19^׀^N, 78º 29^׀^E) during wet season (*Kharif*)—2016. Corresponding maintainer (B) lines were used to denote the CMS lines. The list of hybrid crosses, parental lines and checks are represented in Table 1.

The experiment was executed with two diverse crop cultivation / management methods *i.e.*, irrigated (Conventional puddle transplanted condition) and aerobic (dry direct seeded rice) conditions. The experimental plot was laid out in randomized complete blocks design (RCBD) with three replications. Each plot (both under aerobic and irrigated method) consisted of 96 hills (24 hills × 4 rows) with 20 cm inter-row spacing and 15 cm intra-row spacing and no alley rows between two genotypes.

Soil characteristics of irrigated and aerobic plots were analysed and presented in Table 2. Two sets of seeds were treated with Carbendazim 50% WP, of which one set was allowed to sown manually under aerobic method adopting one-two dry seeds per hill. Immediately after sowing, need based supplementary irrigation were ensured. The second set was raised on the same day in raised nursery bed and 25 days old seedlings were transplanted in main field. Recommended dosages of fertilizers were applied to the main field under irrigated and aerobic conditions. Nitrogen (Urea@46.5% N) at the rate of 100 kg.ha^-1^ was applied in three equal split dosages (at basal, maximum tillering and panicle initiation stages). Phosphorus @ 60 kg.ha^-1^, Potassium @ 40 kg.ha^-1^ and Zinc @25 kg.ha^-1^ were applied as a single dose in the dry soil before final ploughing in the aerobic condition and the similar fertilizer management was also followed in irrigated conditions. One foliar spray of Fe (FeSO_4_@1%) and Zn (ZnSO_4_@2%) was carried out to overcome deficiency at seedling stage (25 DAS) under aerobic condition. Required weed management and plant protection measures were undertaken for healthy crop production. Days to 50% flowering was recorded on plot basis. Three hills in all the genotypes from each replication were randomly tagged and harvested at physiological maturity stage. The samples were sun dried and the moisture content was reduced to 14%. Observations were recorded for key agronomic traits associated with yield viz*.,* Single Plant Yield (SPY) and 1000-grain weight (TGW). The grains were stored at room temperature for estimation of Fe and Zn concentration.

### Grain Fe and Zn analysis

Around 20 g of paddy from each sample was de-husked in non-iron & non-zinc de-huller and the brown rice was cleaned thoroughly using soft tissue paper and transferred to a fresh brown cover^56^. Grain Fe and Zn concentration in 5 g of unpolished rice (brown rice) were estimated^57^ by Energy Dispersive X-Ray Fluorescence Spectrometry (ED-XRF, Model- X-Supreme 8000). This instrument is quite useful in non-destructive determination of relative Fe and Zn concentrations in rice samples with more ease.

### Statistical analysis

The data recorded on studied traits in parents and hybrids were subjected to analysis of variance (ANOVA) using a general linear model as implemented in SAS version 9.1 (SAS Institute, Cary, NC, USA). In addition, variances attributed to general combining ability (GCA) (σ^2^ GCA) and specific combining ability (SCA) (σ^2^ SCA) were calculated to estimate the predictability ratio [2 σ^2^ GCA / (2σ^2^ GCA + σ^2^ SCA)]. Correlations among different traits were made based on Pearson’s product-moment correlation as executed in R (R Development Core Team 2008).

### Estimation of heterosis and combining ability

For each hybrid cross combination, the standard heterosis (SH), mid-parent heterosis (MPH) and better parent heterosis (BPH) were estimated^58,59^. The means of the check – CR Dhan 202 (C1) was used for estimation of standard heterosis (SH) as it is the best-performing national check in aerobic trial of All India Coordinated Rice Improvement Programme (AICRIP, http://www.aicrip-intranet.in/). Heterosis levels were calculated using the formulae of Gramerji^14^.

The significance of calculated heterosis levels were evaluated using the t test^60^. Estimated t values were compared with two-tailed tabular t values with corresponding error degrees of freedom (*df*) at the 95 and 99% confidence intervals (CI). GCA and SCA effects and their variances were calculated using Line × Tester (L × T) design proposed by Kempthorne^55^ using TNAUSTAT statistical package^61^. GSCA was defined as the sum of GCAs for two parents of a cross combination. The GSCA conception in heterosis breeding was made by Huang^18^. This can be considered as an alternative to SCA due to its advantage by relying on GCA values.

## Supplementary Information

Supplementary Information.
