# Supplementary material for: Grain Fe and Zn content, heterosis, combining ability and its association with grain yield in irrigated and aerobic rice
Source: Sci Rep. 2021 May 19;11:10579. doi: 10.1038/s41598-021-90038-4 (PMC8134482; doi:10.1038/s41598-021-90038-4)
Supplement: Supplementary file 1 — Supplementary Information. [file 41598_2021_90038_MOESM1_ESM.pdf]

Grain Fe and Zn content, Heterosis, Combining ability and its association with grain yield in irrigated and aerobic rice

Anusha G[1], Sanjeeva Rao D[1], Jaldhani V[1], Beulah P[1], Neeraja CN[1], Gireesh C[1], Anantha MS[1], Suneetha K[1], Santhosha R[1], Hari Prasad AS[1], Sundaram RM[1], Sheshu Madhav M[1], Fiyaz A[1], Brajendra P[1], Tuti MD[1], Bhave MHV[2], Radha Krishna KV[2], Ali J[3], Subrahmanyam D[1] and Senguttuvel P[1*]

1. Indian Institute of Rice Research (ICAR-IIRR), Hyderabad, India

2. Professor Jaya Shankar Telangana State Agricultural University (PJTSAU), Hyderabad, India

3. International Rice Research Institute (IRRI), Los Baños, Laguna 4031, Philippines

*Corresponding author: senguttuvel@gmail.com

Supplementary Table 1. Estimation of relative GSCA (General Sum of Combining Ability) effects of crosses for yield components and grain Fe and Zn concentration in irrigated and aerobic methods.

	Irrigated				Aerobic			
**Entry**	**TGW**	**SPY**	**FE**	**ZN**	**TGW**	**SPY**	**FE**	**ZN**
H1 (L1 × T1)	-2.37	-1.94	0.46	2.74	-0.06	-2.71	-0.54	-5.18
H2 (L1 × T2)	-1.08	-1.35	0.62	0.2	1.6	-1.91	-0.53	1.52
H3 (L1 × T3)	-2.46	-1.28	0.01	0.64	0.95	-0.79	-1.4	-1.18
H4 (L1 × T4)	-0.37	-1.06	-0.01	-0.74	1.28	-1.43	-0.57	-2.2
H5 (L2 × T1)	0.2	-0.76	0.28	2.54	-1	-1.17	0.34	-3.44
H6 (L2 × T2)	1.49	-0.17	0.44	0	0.66	-0.37	0.35	3.26
H7 (L2 × T3)	0.11	-0.1	-0.17	0.44	0.01	0.75	-0.52	0.56
H8 (L2 × T4)	2.2	0.12	-0.19	-0.94	0.34	0.11	0.31	-0.46
H9 (L3 × T1)	0.71	0.85	-0.12	1.39	0.29	0.49	0.25	-3.21
H10 (L3 × T2)	2	1.44	0.04	-1.15	1.95	1.29	0.26	3.49
H11 (L3 × T3)	0.62	1.51	-0.57	-0.71	1.3	2.41	-0.61	0.79
H12 (L3 × T4)	2.71	1.73	-0.59	-2.09	1.63	1.77	0.22	-0.23
H13 (L4 × T1)	-1.75	-0.27	0.13	1.45	-1.34	-0.62	0.83	-1.85
H14 (L4 × T2)	-0.46	0.32	0.29	-1.09	0.32	0.18	0.84	4.85
H15 (L4 × T3)	-1.84	0.39	-0.32	-0.65	-0.33	1.3	-0.03	2.15
H16 (L4 × T4)	0.25	0.61	-0.34	-2.03	0	0.66	0.8	1.13

TGW - 1000 Grain Weight; SPY – Single Plant Yield; FE – Grain Fe content; ZN – Grain Zn content; H1 to H16 indicates the Hybrids.

Supplementary Table 2. Pearson's correlation between phenotype, heterosis and combining ability effects for yield components (TGW and SPY), grain Fe and Zn concentrations derived from line × tester mating design in irrigated and aerobic methods.

	**IRRIGATED**				**AEROBIC**			
	**TGW**	**SPY**	**FE**	**ZN**	**TGW**	**SPY**	**FE**	**ZN**
**GSCA Versus**								
GCA	0.17	0.36	0.85**	0.84**	0.53	0.65*	0.25	0.74*
SCA	0.38	0.73**	-0.60*	-0.57*	-0.13	0.73**	0.66**	0.49*
SH	0.37	0.60*	-0.65**	-0.58*	-0.15	0.69**	0.68**	0.49*
MPH	0.37	0.66**	-0.59*	-0.59*	-0.14	0.68**	0.68**	0.45*
BPH	0.36	0.68**	-0.61*	-0.59*	-0.14	0.63**	0.66**	0.44*
Phenotype	-0.40	-0.32	0.23	0.06	0.07	-0.16	-0.29	0.53*
**GCA Versus**								
SCA	0.27	0.54	-0.43	-0.53	0.43	0.43	0.25	0.25
SH	0.33	0.60	-0.45	-0.25	0.40	0.36	0.12	0.25
MPH	0.27	0.62*	-0.38	-0.37	0.35	0.56	0.10	0.20
BPH	0.28	0.37	-0.34	-0.44	0.39	0.38	0.32	0.26
Phenotype	-0.48	-0.18	0.08	-0.02	0.07	-0.30	0.54	0.11
**SCA Versus**								
SH	0.95***	0.93***	0.94***	0.95***	0.99***	0.90***	0.98***	1.00***
MPH	0.97***	0.95***	0.92***	0.89***	0.99***	0.94***	0.99***	0.97***
BPH	0.96***	0.93***	0.96***	0.84***	0.96***	0.90***	0.99***	0.97***
Phenotype	-0.12	-0.10	-0.31	0.28	-0.21	-0.02	-0.27	0.54*

GSCA - General Sum of Combining Ability; GCA – General Combining Ability; SCA – Specific Combining Ability; SH – Standard Heterosis; MPH – Mid-Parent Heterosis; BPH – Best-Parent Heterosis; TGW - 1000 Grain Weight; SPY – Single Plant Yield; FE – Grain Fe content; ZN – Grain Zn content. $^*P < 0.05$; $^{**}P < 0.01$; $^{***}P < 0.001$

Supplementary Table 3. Pearson's correlation among traits in parents and hybrids in irrigated and aerobic methods. * Significant at the 0.05 probability level; Correlation coefficients among traits in hybrids (above diagonal) and parents (below diagonal); Values outside the parentheses are from irrigated method and values within the parentheses are from aerobic method.

**TGW**	0.29 (0.51*)	-0.15 (-0.11)	-0.31 (0.36)
0.73* (0.62*)	**SPY**	-0.23 (0.43*)	-0.05 (0.44*)
0.33 (0.62)	0.44 (0.38)	**FE**	0.50* (0.26)
0.49 (-0.49)	0.37 (-0.26)	0.38 (-0.73*)	**ZN**

TGW - 1000 Grain Weight; SPY – Single Plant Yield; FE – Grain Fe content; ZN – Grain Zn content

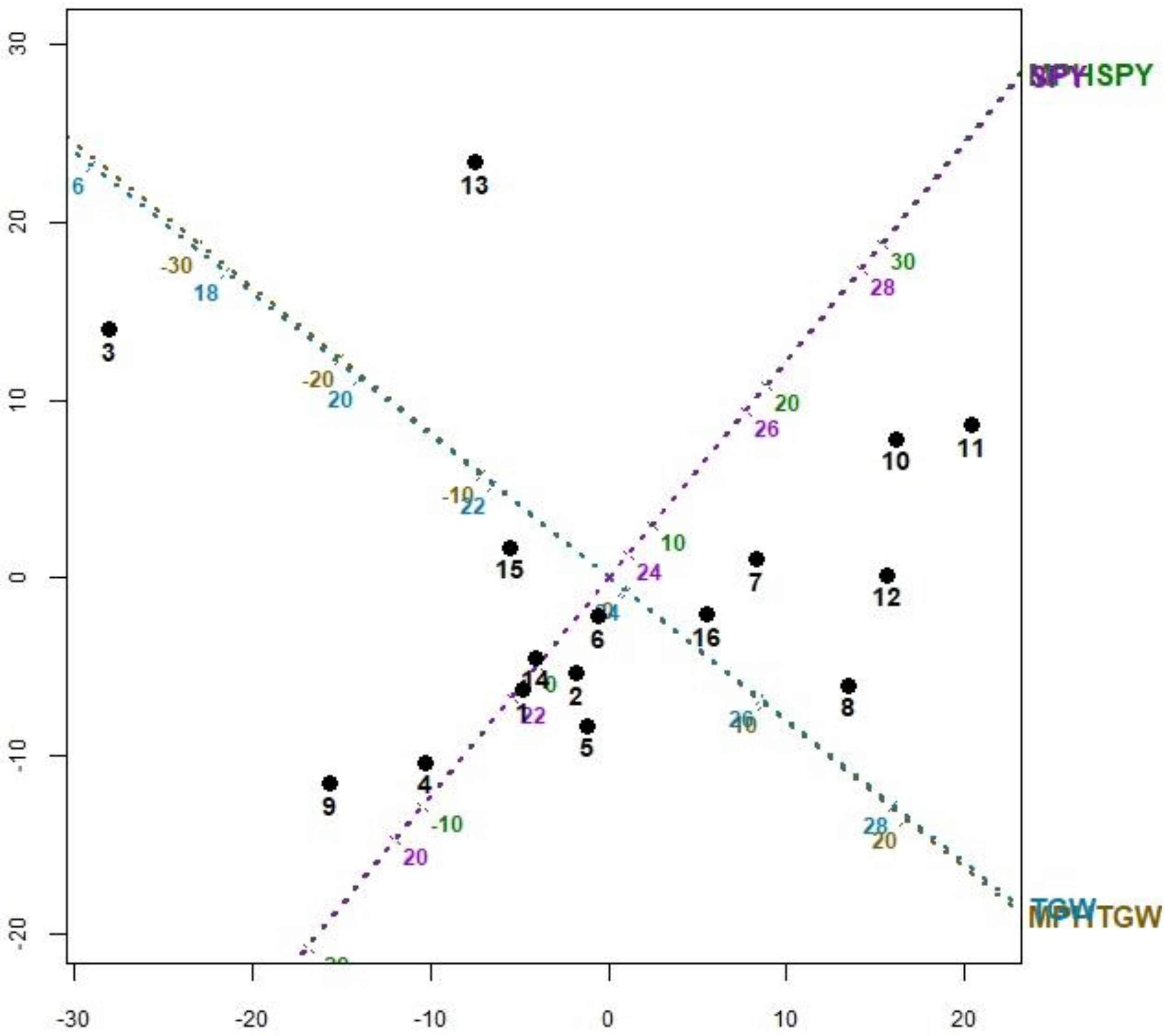

Supplementary figure 1. Principal component analysis (PCA) biplot diagrams for crosses representing Mid-Parent Heterosis (MPH) and mean values of TGW (1000 Grain Weight) and SPY (Single Plant Yield) in Irrigated method. 1 to 16 indicates the hybrids H1 to H16.

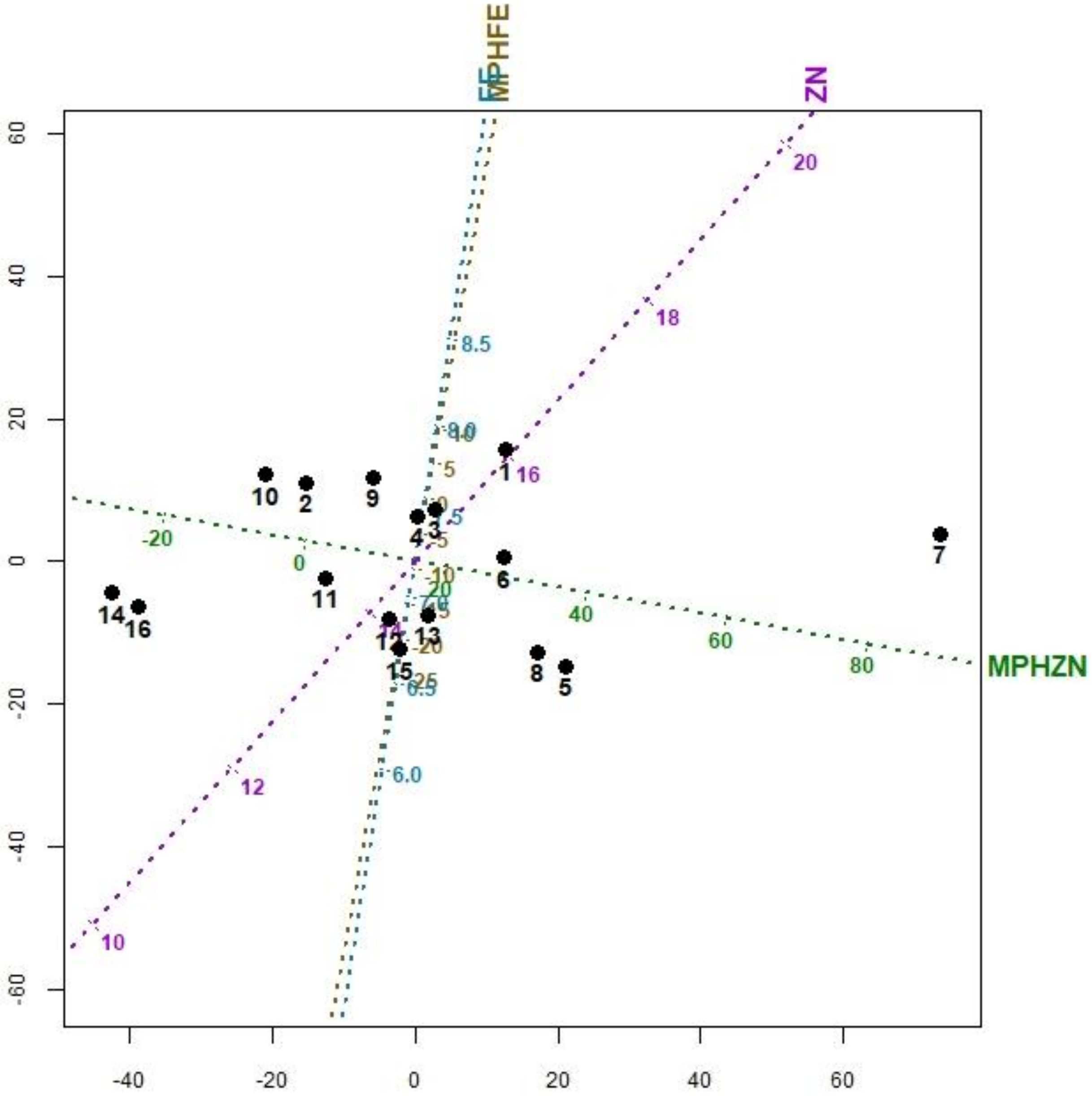

Supplementary figure 2. Principal component analysis (PCA) biplot diagrams for crosses representing Mid-Parent Heterosis (MPH) and mean values of Grain Zn and Grain Fe content in Irrigated method. 1 to 16 indicates the Hybrids H1 to H16.

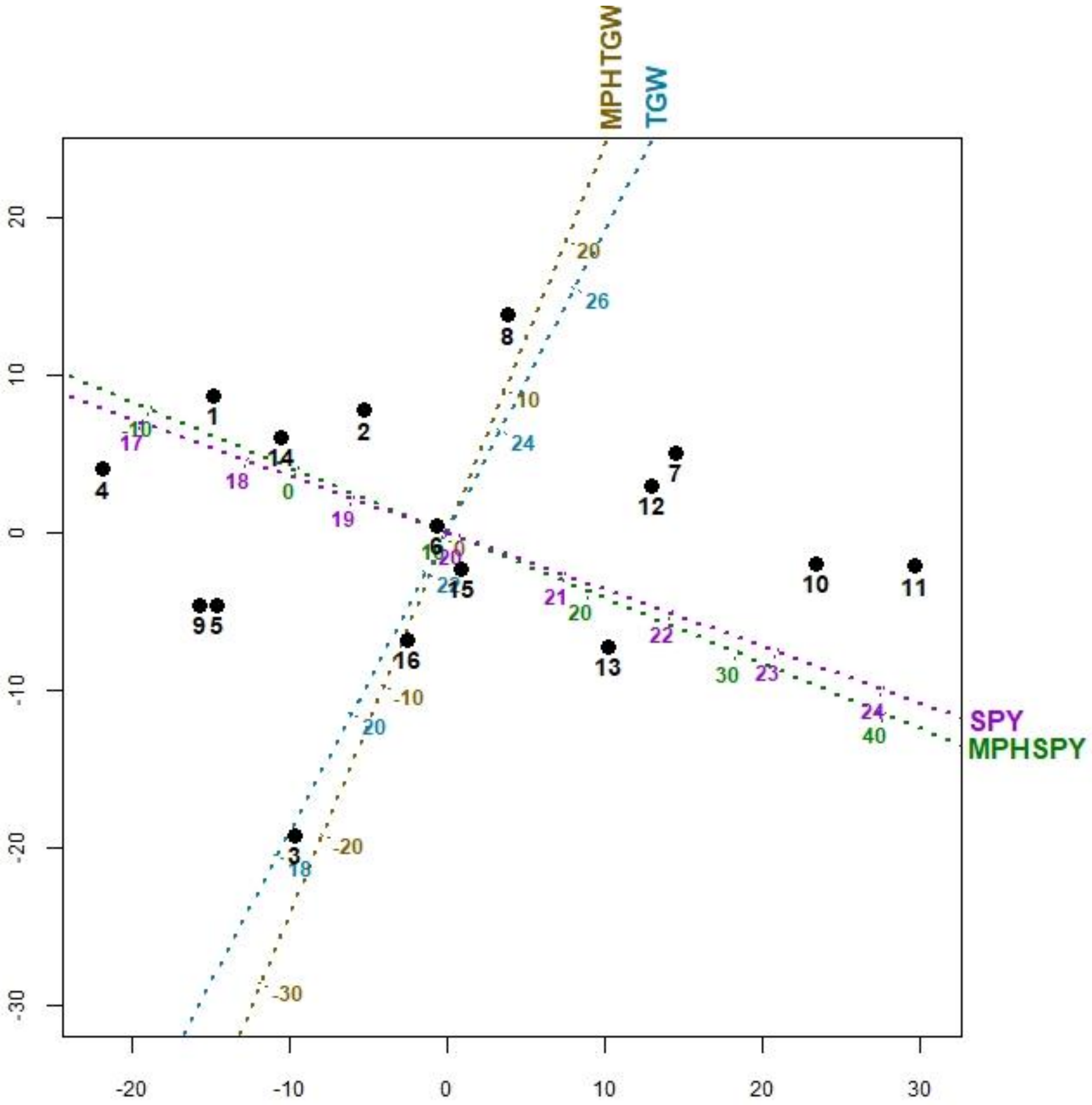

Supplementary figure 3. Principal component analysis (PCA) biplot diagrams for crosses representing Mid-Parent Heterosis (MPH) and mean values of TGW (1000 Grain Weight) and SPY (Single Plant Yield) in Aerobic method. 1 to 16 indicates the Hybrids H1 to H16.

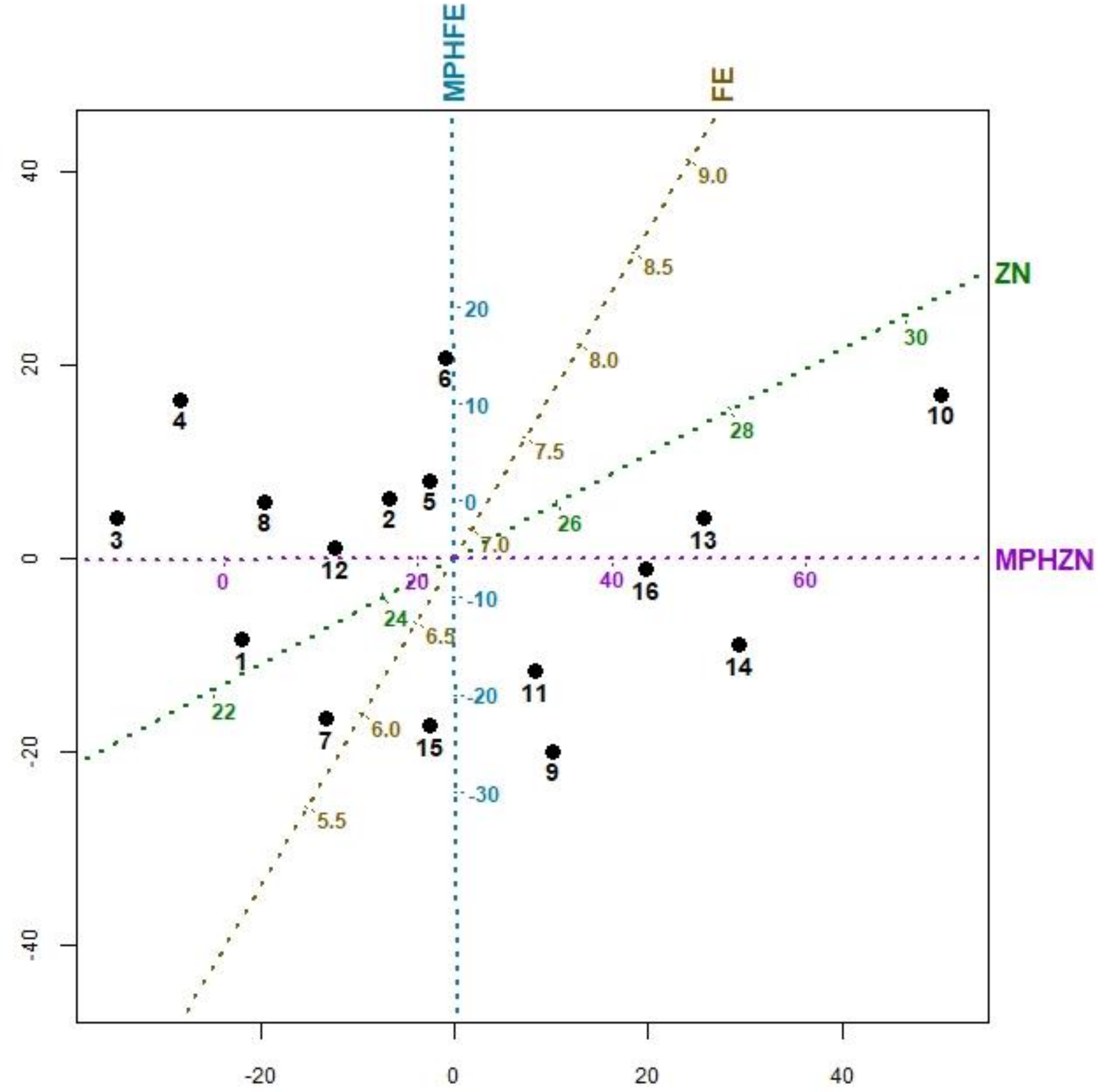

Supplementary figure 4. Principal component analysis (PCA) biplot diagrams for crosses representing Mid-Parent Heterosis (MPH) and mean values of Grain Fe and Grain Zn content in Aerobic method. 1 to 16 indicates the Hybrids H1 to H16.

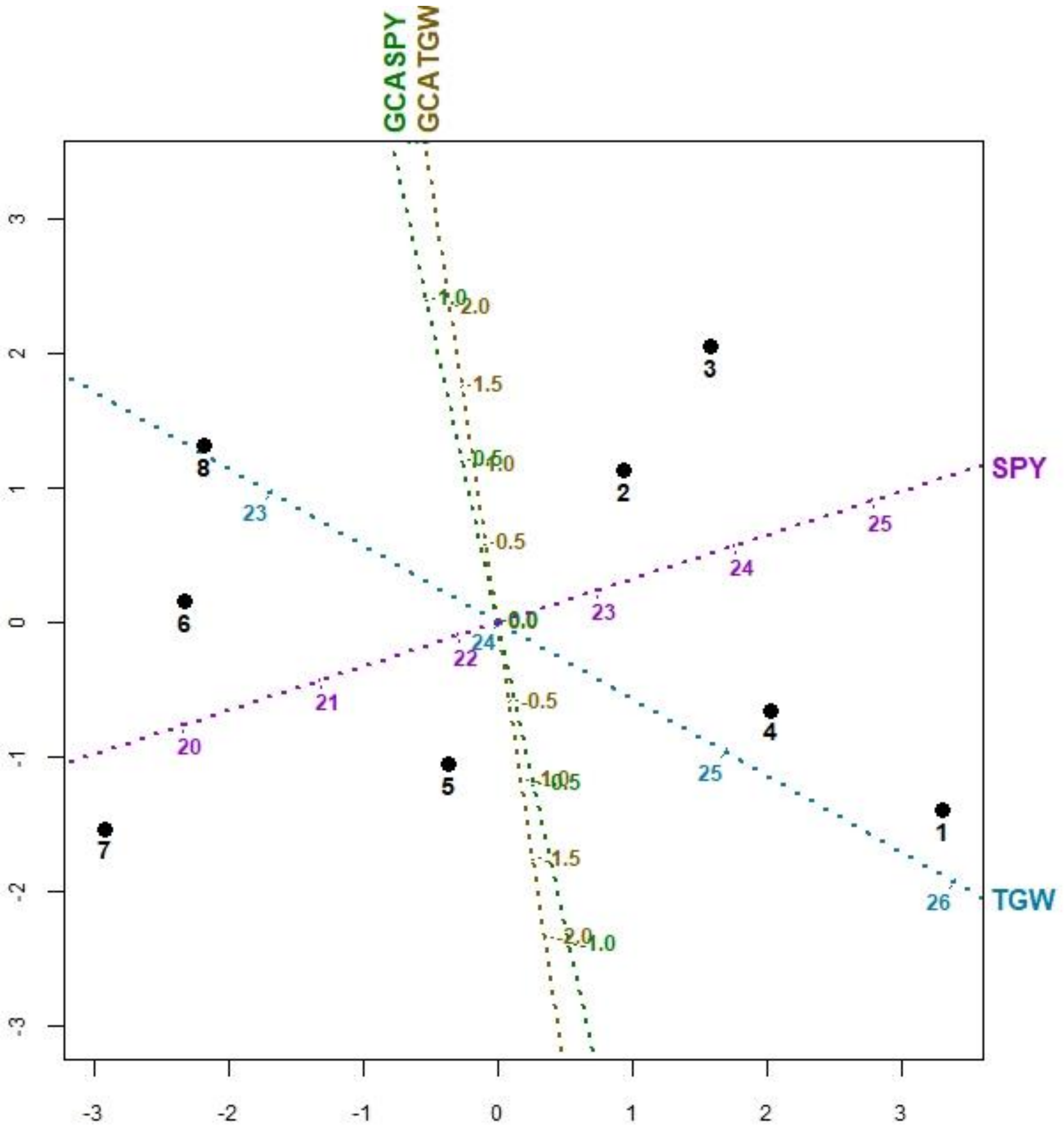

Supplementary figure 5. Principal component analysis (PCA) biplot diagrams for parents representing General Combining Ability (GCA) and mean values of TGW (1000 Grain Weight) and SPY (Single Plant Yield) in Irrigated method. 1 to 4 indicates the Lines L1 to L4; 5 to 8 indicates the Testers T1 to T4.

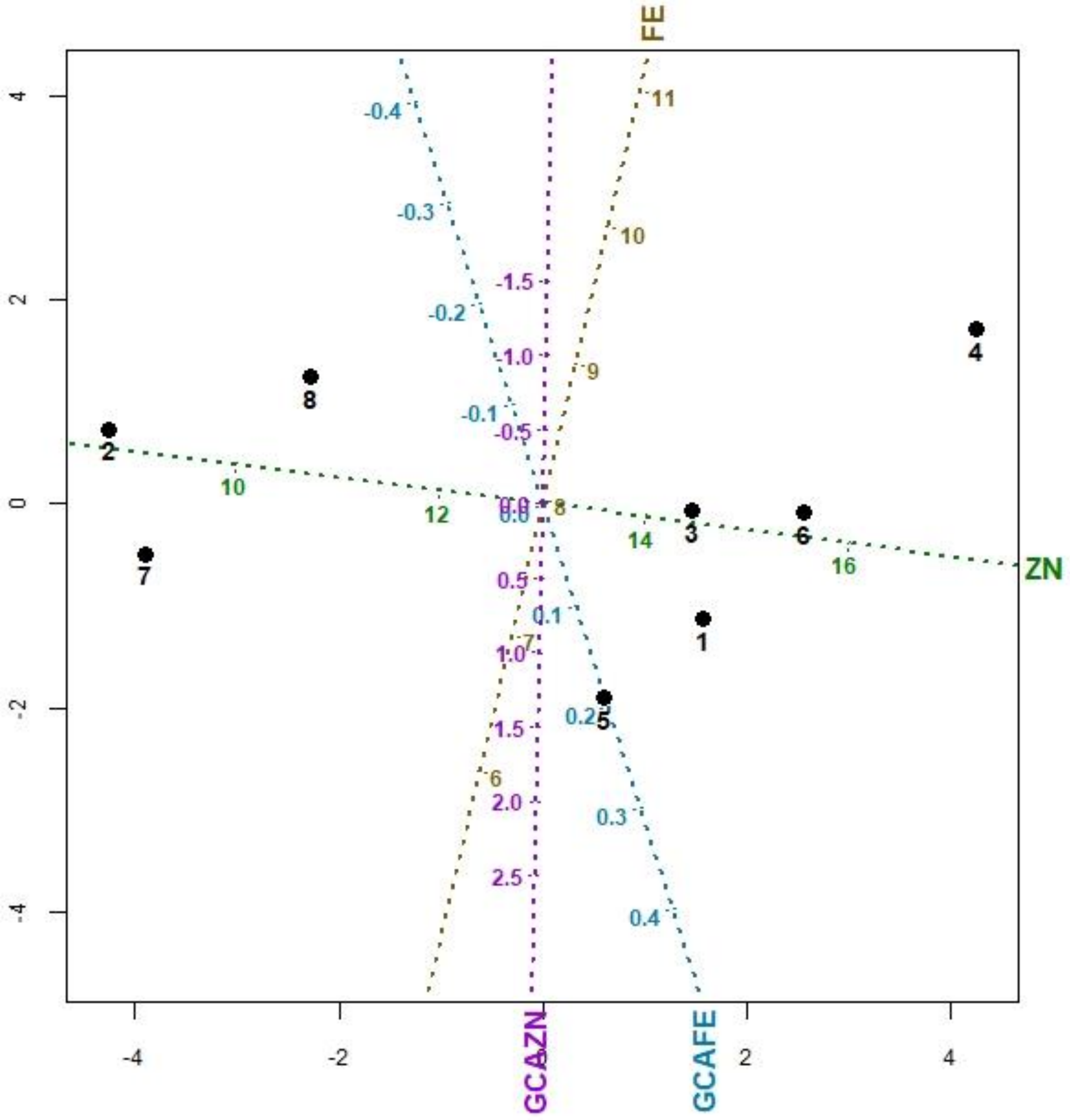

Supplementary figure 6. Principal component analysis (PCA) biplot diagrams for parents representing General Combining Ability (GCA) and mean values of Grain Fe and Grain Zn content in Irrigated method. 1 to 4 indicates the Lines L1 to L4; 5 to 8 indicates the Testers T1 to T4.

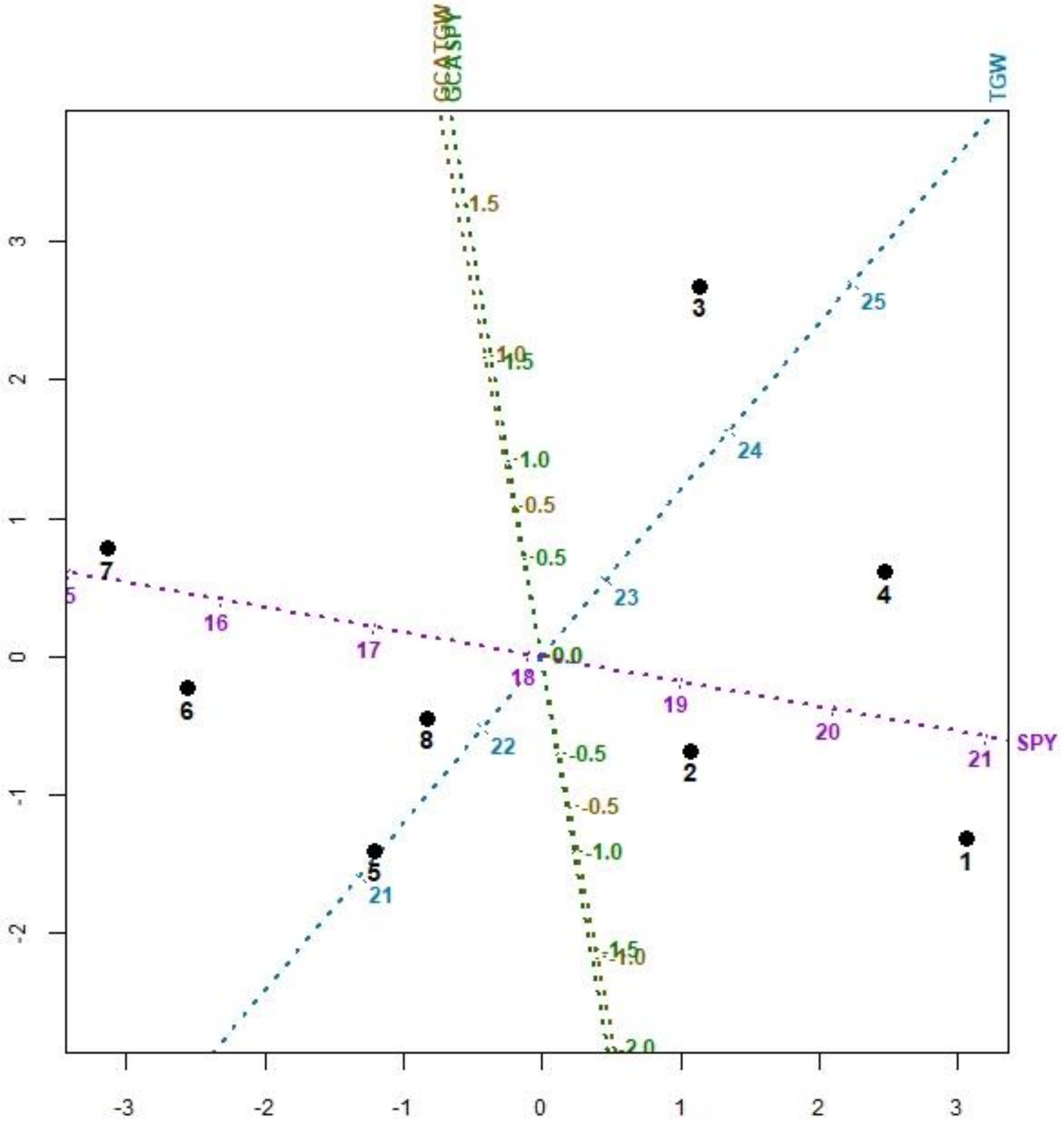

Supplementary figure 7. Principal component analysis (PCA) biplot diagrams for parents representing General Combining Ability (GCA) and mean values of TGW (1000 Grain Weight) and SPY (Single Plant Yield) in Aerobic method. 1 to 4 indicates the Lines L1 to L4; 5 to 8 indicates the Testers T1 to T4.

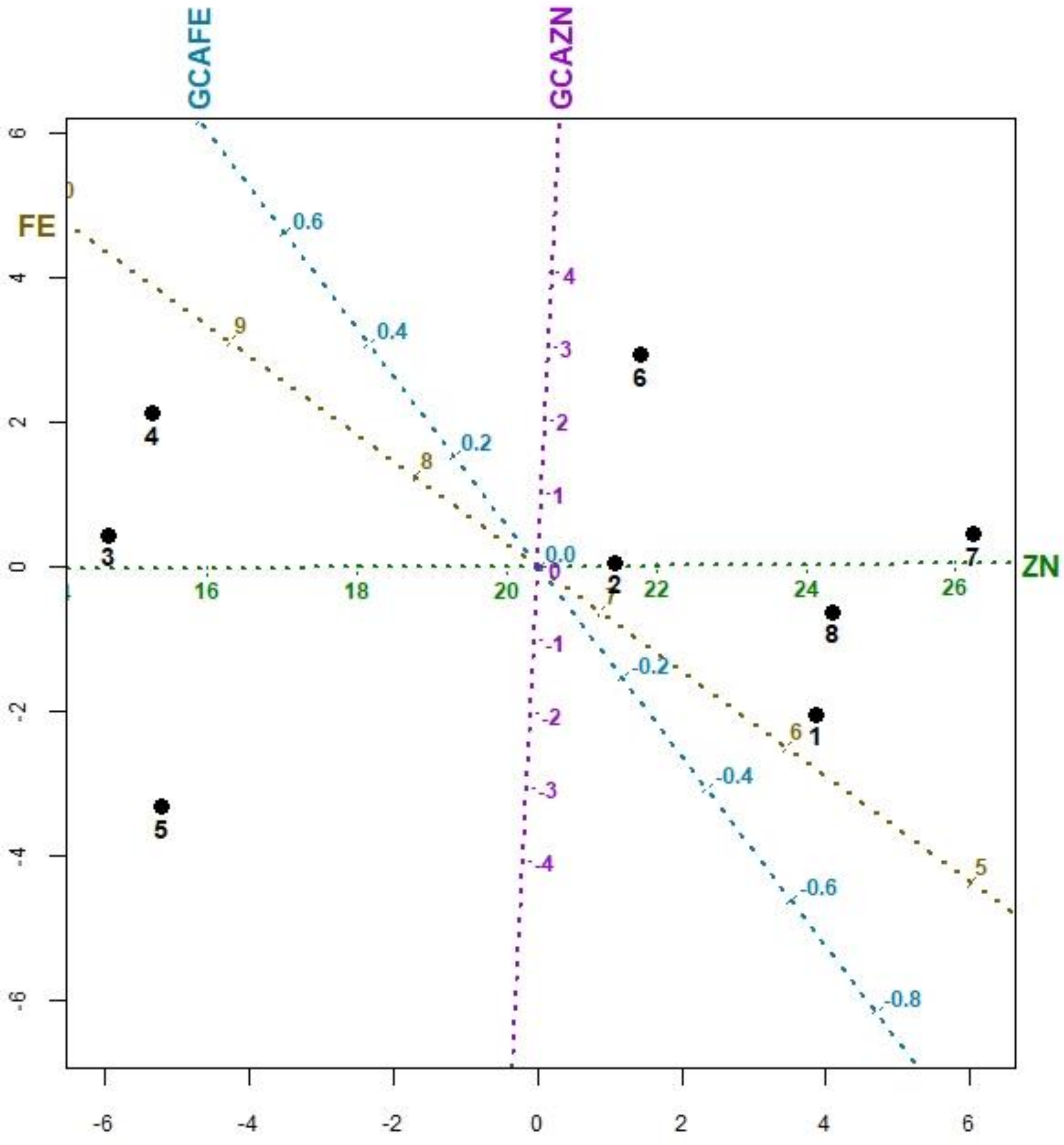

Supplementary figure 8. Principal component analysis (PCA) biplot diagrams for parents representing General Combining Ability (GCA) and mean values of Grain Fe and Grain Zn content in Aerobic method. 1 to 4 indicates the Lines L1 to L4; 5 to 8 indicates the Testers T1 to T4.

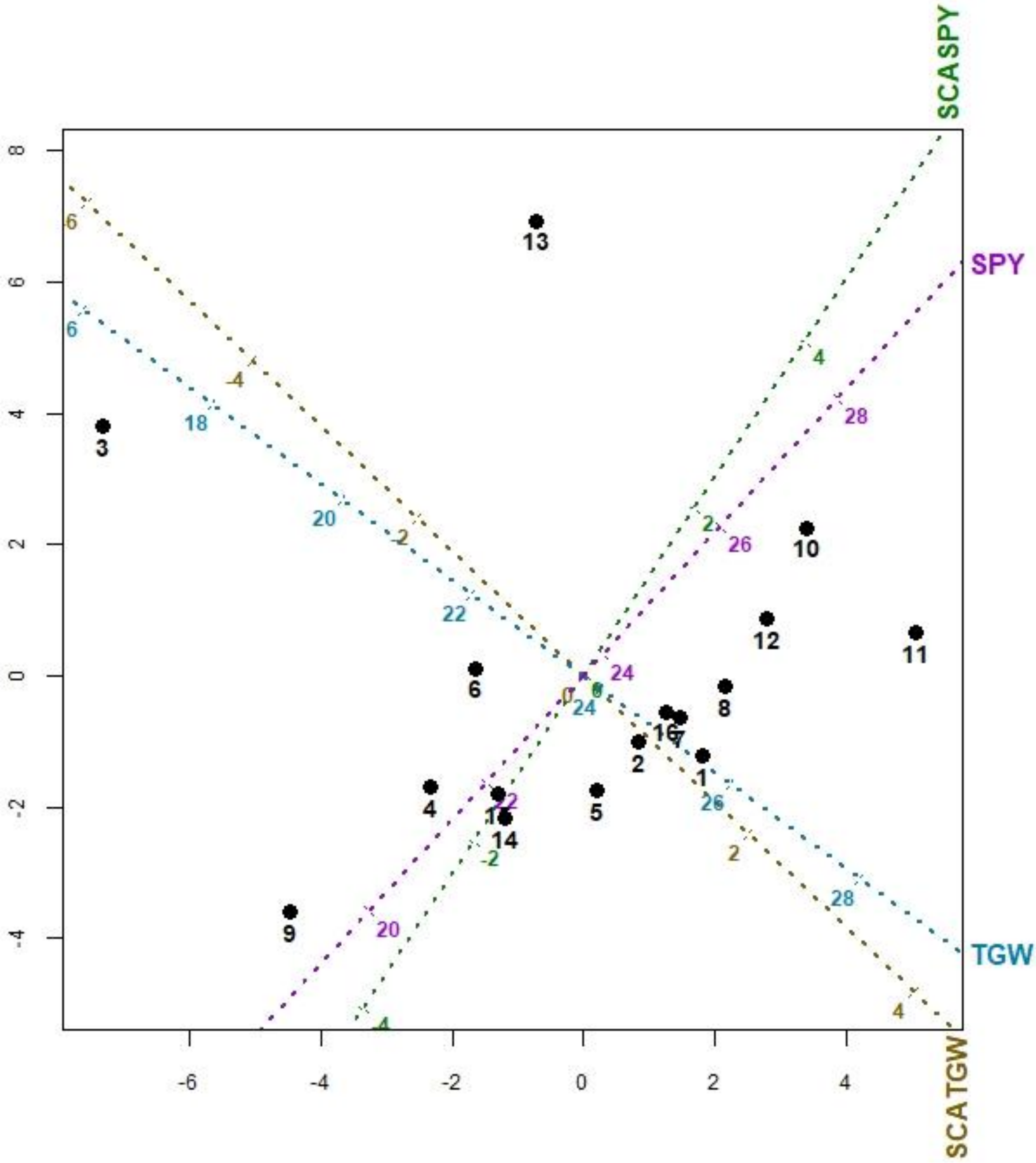

Supplementary figure 9. Principal component analysis (PCA) biplot diagrams for crosses representing Specific Combining Ability (SCA) and mean values of TGW (1000 Grain Weight) and SPY (Single Plant Yield) in Irrigated method. 1 to 16 indicates the Hybrids H1 to H16.

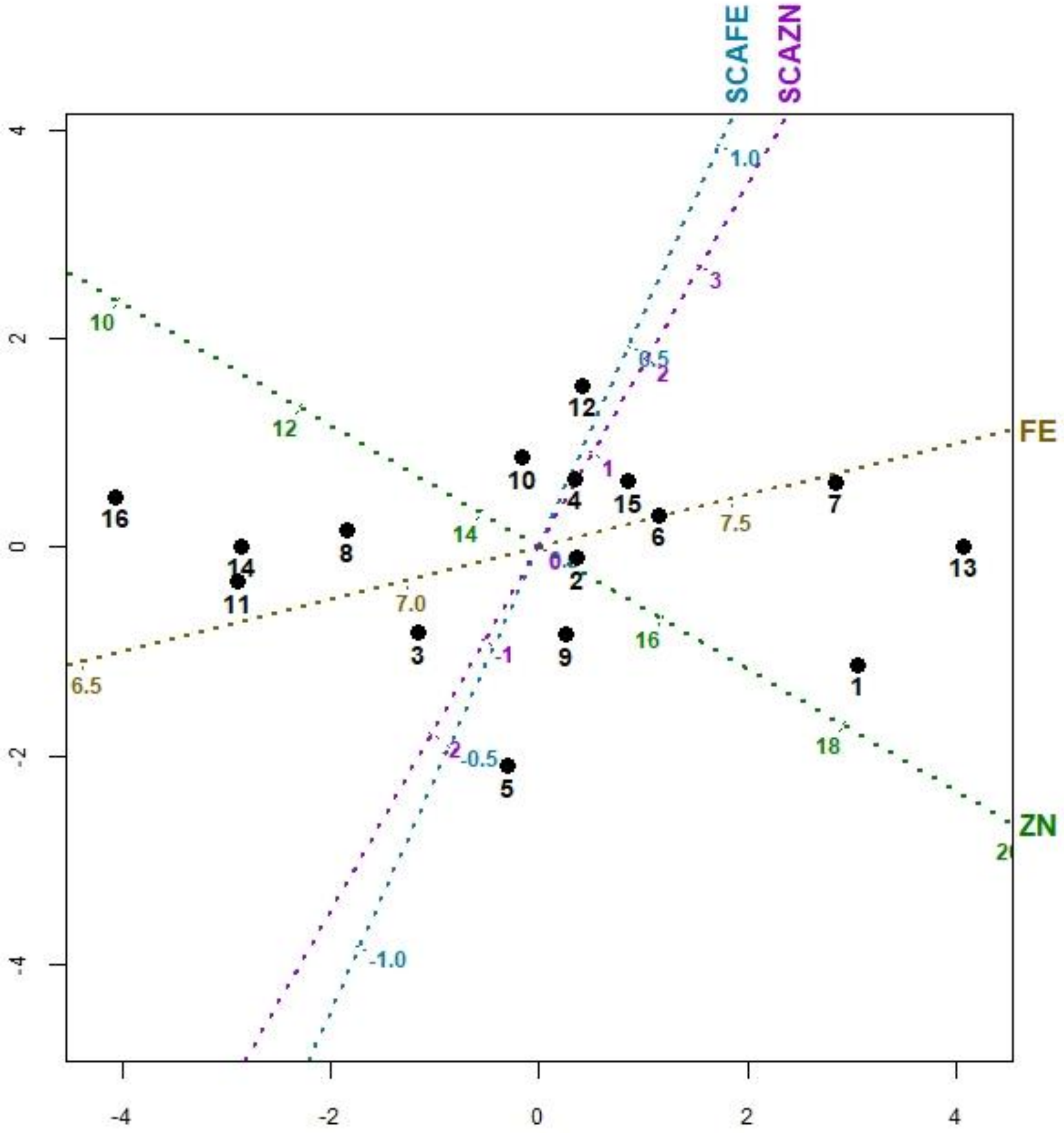

Supplementary figure 10. Principal component analysis (PCA) biplot diagrams for crosses representing Specific Combining Ability (SCA) and mean values of Grain Fe and Grain Zn content in Irrigated method. 1 to 16 indicates the Hybrids H1 to H16.

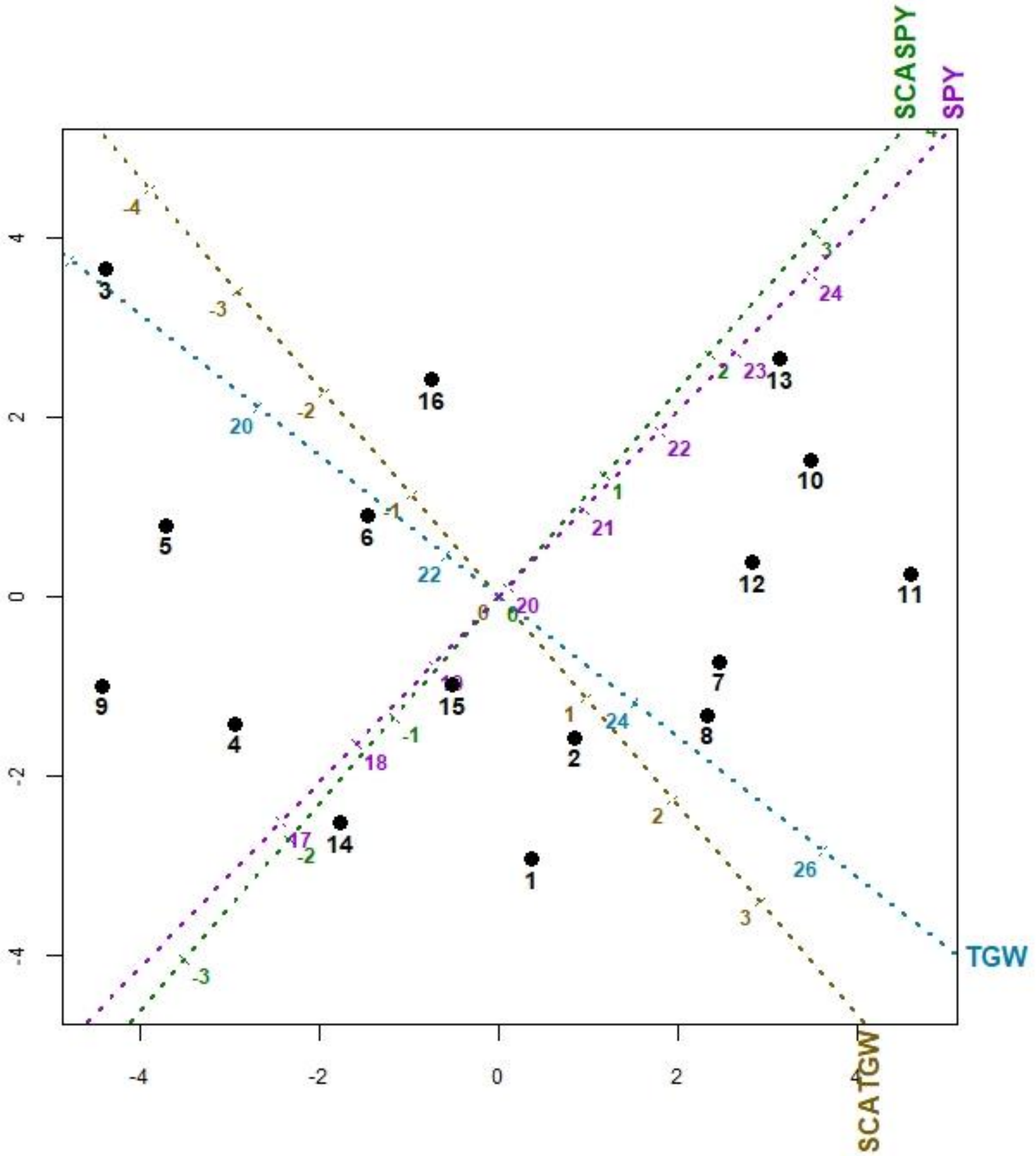

Supplementary figure 11. Principal component analysis (PCA) biplot diagrams for crosses representing Specific Combining Ability (SCA) and mean values of TGW (1000 Grain Weight) and SPY (Single Plant Yield) in Aerobic method. 1 to 16 indicates the Hybrids H1 to H16.

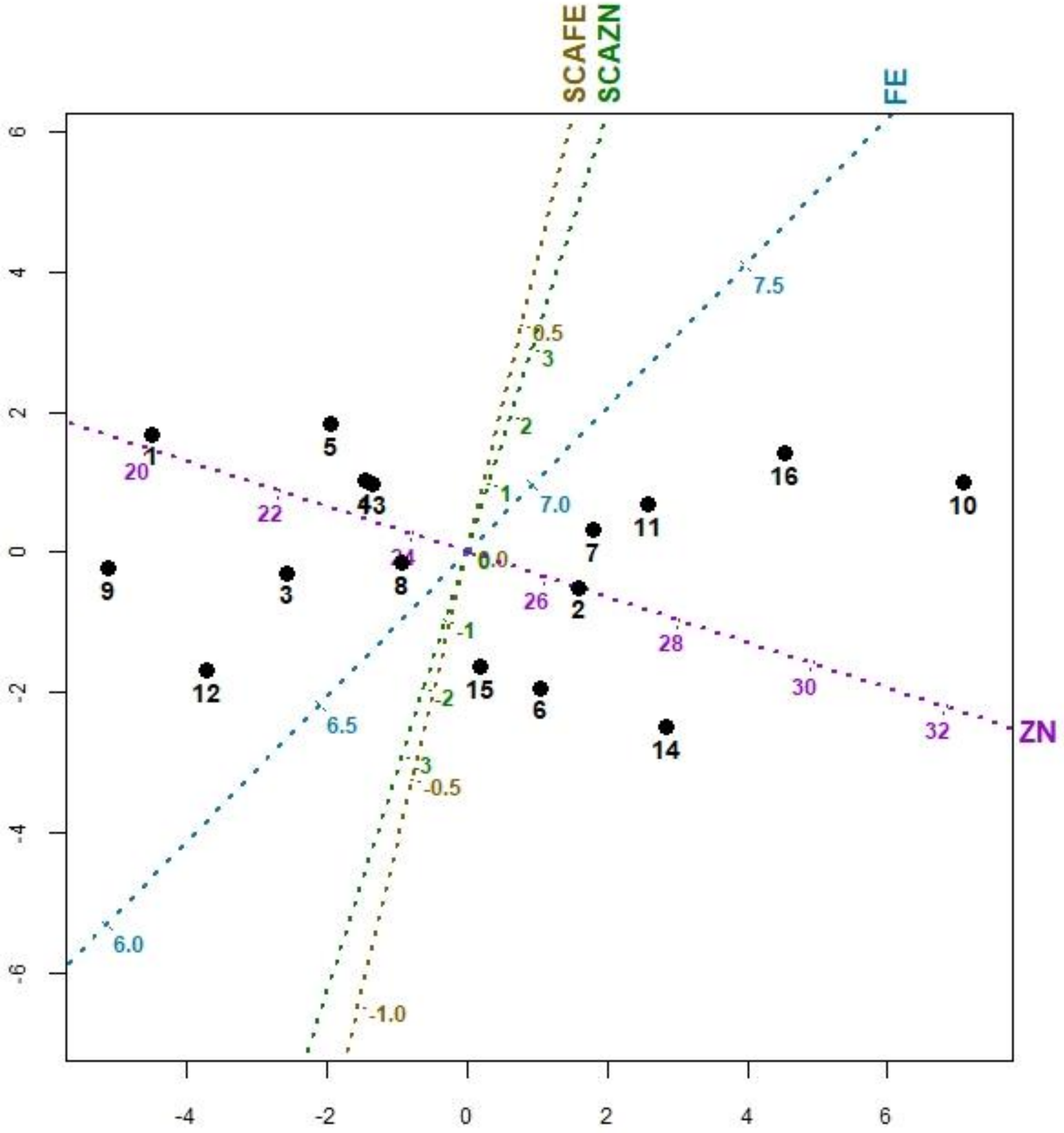

Supplementary figure 12. Principal component analysis (PCA) biplot diagrams for crosses representing Specific Combining Ability (SCA) and mean values of Grain Fe and Grain Zn content in Aerobic method. 1 to 16 indicates the Hybrids H1 to H16.
